# Production of Highly Uniform Midbrain Organoids from Human Pluripotent Stem Cells

**DOI:** 10.1155/2023/3320211

**Published:** 2023-09-29

**Authors:** Xuerui Yao, Ji Hyun Kang, Kee-Pyo Kim, Hyogeun Shin, Zhe-Long Jin, Hao Guo, Yong-Nan Xu, Ying-Hua Li, Sai Hali, Jeongwoo Kwon, Hyeonwoo La, Chanhyeok Park, Yong-June Kim, Lin Wang, Kwonho Hong, Qilong Cao, Il-Joo Cho, Nam-Hyung Kim, Dong Wook Han

**Affiliations:** ^1^Guangdong Provincial Key Laboratory of Large Animal Models for Biomedicine, School of Biotechnology and Health Sciences, Wuyi University, Jiangmen, China; ^2^International Healthcare Innovation Institute (Jiangmen), Jianghai, Jiangmen, Guangdong Province, China; ^3^Research and Development Department, Qingdao Haier Biotech Co. Ltd., Qingdao, China; ^4^Laboratory of Stem Cells and Organoids, OrganFactory Co. Ltd., Cheongju 28864, Republic of Korea; ^5^Department of Life Sciences, College of Medicine, The Catholic University of Korea, Seoul 06591, Republic of Korea; ^6^Center for BioMicrosystems, Brain Science Institute, Korea Institute of Science and Technology (KIST), Seoul, Republic of Korea; ^7^Division of Bio-Medical Science and Technology, KIST School, Korea University of Science and Technology (UST), Daejeon, Republic of Korea; ^8^Institute of Ophthalmology, University College London, London, UK; ^9^Primate Resources Center, Korea Research Institute of Bioscience and Biotechnology, Jeongeup, Republic of Korea; ^10^Department of Stem Cell and Regenerative Biotechnology, The Institute of Advanced Regenerative Science, Konkuk University, Seoul 05029, Republic of Korea; ^11^Department of Urology, College of Medicine, Chungbuk National University, Cheongju, Republic of Korea; ^12^Department of Urology, Chungbuk National University Hospital, Cheongju, Republic of Korea

## Abstract

Brain organoids have been considered as an advanced platform for *in vitro* disease modeling and drug screening, but numerous roadblocks exist, such as lack of large-scale production technology and lengthy protocols with multiple manipulation steps, impeding the industrial translation of brain organoid technology. Here, we describe the high-speed and large-scale production of midbrain organoids using a high-throughput screening-compatible platform within 30 days. Micro midbrain organoids (*µ*MOs) exhibit a highly uniform morphology and gene expression pattern with minimal variability. Notably, *µ*MOs show dramatically accelerated maturation, resulting in the generation of functional *µ*MOs within only 30 days of differentiation. Furthermore, individual *µ*MOs display highly consistent responsiveness to neurotoxin, suggesting their usefulness as an *in vitro* high-throughput drug toxicity screening platform. Collectively, our data indicate that *µ*MO technology could represent an advanced and robust platform for *in vitro* disease modeling and drug screening for human neuronal diseases.

## 1. Introduction

Drug discovery is a high-risk but high-return endeavor, with the vast majority of drug candidates failing to demonstrate efficacy or being associated with unexpected toxicity during preclinical and clinical trial testing [1]. Additionally, many drug candidates exhibit a lack of efficacy during clinical trials despite demonstrating therapeutic effects in animal models [2, 3], marking interspecies differences as another hurdle to overcome for efficient drug discovery. Induced pluripotent stem cell (iPSC) technology [4] has recently been considered as a useful tool for not only reproducing patient-specific pathophysiology [5, 6] but also minimizing the use of preclinical animal models, potentially enabling highly efficient and cost-effective patient-specific drug development [7]. However, 2D cell culture-based iPSC technology is associated with another critical problem: the incomplete recapitulation of disease pathophysiology due to its limited capacity for reproducing both the *in vivo* niche and cellular interactions [8, 9].

Organoid technology, a recent breakthrough in the stem cell field, has been highlighted as the most innovative stem cell technology for understanding pathological developmental processes and the mechanisms underlying various diseases [10]. Indeed, recent studies using organoid technology have unveiled the mechanism of several diseases including the infamous coronavirus 2019 (COVID-19) disease and have discovered novel therapeutic candidates by closely recapitulating disease-specific pathophysiology at the organ level [11]. Notwithstanding, industrial translation of organoid technology, including brain organoids, has been hampered by multiple technical roadblocks such as: (1) lack of proper culture technology for producing adult organ-like mature organoids [12, 13]; (2) biased cellular composition [14, 15]; (3) low efficiency and reproducibility [16, 17]; (4) variability among individual organoids both within and across batches [16]; and (5) frequently observed uncontrolled differentiation, leading to outgrowths [18, 19], and necrotic dead core caused by insufficient delivery of oxygen and nutrients to the organoid core due to lack of a vascular system [20, 21]. Moreover, the process of generating relatively mature brain organoids takes at least 3–6 months and involves multiple manipulation steps such as seeding the starting cells, embryoid body formation, embedding into the extracellular matrix, and using an orbital shaker to facilitate organoid maturation [14, 15, 22, 23]. Such a lengthy process with multiple manipulation steps, together with the aforementioned technical roadblocks in brain organoid production, results inevitably in an extremely high cost for producing brain organoids. Therefore, the development of key technologies that could overcome all the hurdles and limitations associated with organoid generation is required for the industrialization of organoid technology toward drug screening for human neuronal diseases.

To facilitate the industrialization of brain organoid technology, the current study aims to develop the high-speed and large-scale standardized production protocol for the generation of midbrain organoids using a high-throughput screening- (HTS-) compatible platform. Under this platform, we were able to generate micro midbrain organoids (*µ*MOs) within 4 weeks of differentiation. Compared with typical midbrain organoids, *µ*MOs exhibit a uniform and consistent morphology, size, and gene expression pattern. Strikingly, *µ*MOs exhibit dramatically accelerated maturation within only 30 days of differentiation, such as the homogeneous distribution of mature dopaminergic neurons, presence of other *in vivo*-like cellular compartments including glial cells, production of neuromelanin-like granules, and mature electrophysiological features. The highly uniform *µ*MOs produced by a high-speed and industry-scale methodology also facilitate *in vitro* drug toxicity screening. Our novel and robust protocol for generating *µ*MOs may represent a key technology for the industrial application of brain organoids such as high-throughput drug screening for Parkinson's disease and other neurodegenerative diseases.

## 2. Results

### 2.1. One-Step Generation of Micro Midbrain Organoids Using a High-Throughput Platform

**Figure 1:**
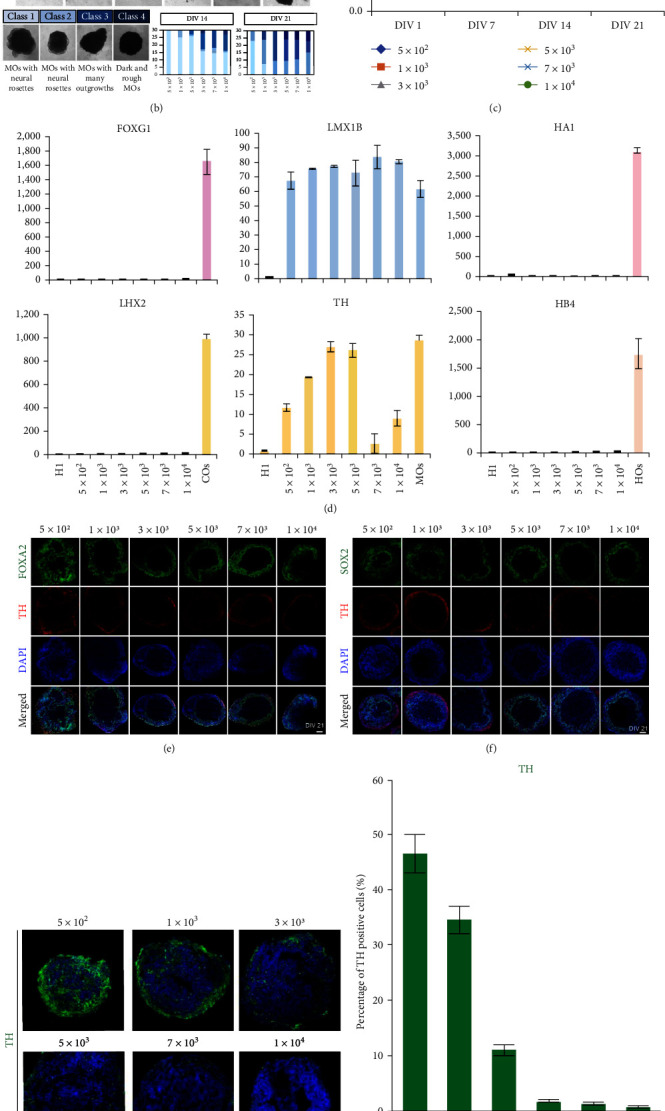
Generation of *µ*MOs using a high-throughput platform. (a) Schematic illustration of the procedure for generating *µ*MOs. The procedures and timelines for generating *µ*MOs and typical MOs are described. (b) The morphology and quality of MOs generated from a different number of starting cells (DIV 14 and 21). Scale bar, 1 mm. (c) Average diameter of MOs generated from a different number of starting cells on DIV 1, 7, 14, and 21 of differentiation. Data are presented as mean ± SD (*n* = 20 for each number of starting cells). (d) Expression of forebrain (FOXG1 and LHX2), midbrain (LMX1B and TH), and hindbrain (HA1 and HB4) specific marker genes in MOs generated from a different number of starting cells was analyzed by qPCR (DIV 14). Expression levels are normalized to those of undifferentiated hESCs. Typical cerebral organoids (COs), MOs, and hindbrain organoids (HOs) were also used for positive controls. Data are presented as mean ± SD of triplicate values. (e and f) Representative confocal images of MOs generated from a different number of starting cells (DIV 21). Scale bar, 100 *μ*m. (g) Representative confocal images of MOs generated from a different number of starting cells expressing TH (DIV 30). Scale bar, 100 *μ*m. (h) Percentage of TH-positive cells in *µ*MOs generated from a different number of starting cells (DIV 30). Data are presented as mean ± SD of triplicate values. (i) The representative section images displaying global enrichment of MAP2 and TH positive cells in *µ*MOs (DIV 30). Scale bar, 100 *μ*m.

To overcome the technical roadblocks associated with brain organoid technology, such as a lengthy procedure with multiple manipulation steps, the lack of a standardized protocol for producing uniform brain organoids with consistent quality, variability in organoid morphology, and other features within and across batches, and the need for a highly scalable production platform, we first tried to establish a one-step simplified protocol for producing midbrain organoids (MOs) using a high-throughput screening- (HTS-) compatible platform such as 96- and 384-well plates. As we used a high-throughput platform for all the MOs generation steps without transferring MOs into other cell culture formats, we sought to optimize the starting cell number (5 × 10^2^, 1 × 10^3^, 3 × 10^3^, 5 × 10^3^, 7 × 10^3^, and 1 × 10^4^) by making slight modifications to our previous MO protocol [23] (Figure 1(a)). We did not make use of the orbital shaker in this study even during the organoid maturation step because a proper flow could not be created in a high-throughput platform using a typical orbital shaker.

We first evaluated the morphology and gene expression patterns of early MOs on DIV (days *in vitro*) 7. MOs generated from a different number of starting cells displayed a uniform morphology but distinct size based on the starting cell number (Figure S1). MOs on DIV 7 showed inactivation of pluripotency markers without activation of mesendodermal genes (Figure S1). In contrast, MOs generated from a different number of starting cells showed activation of ectodermal lineage markers whereas MOs generated from a low number of starting cells (5 × 10^2^ and 1 × 10^3^) showed higher expression of early neuronal markers such as N-CAD, PLZF, SOX2, and FGF8 compared with MOs derived from a high-cell number (7 × 10^3^ and 1 × 10^4^) (Figure S1). This result indicated that MOs generated from 5 × 10^2^ to 1 × 10^3^ starting cells, which have relatively higher surface-to-volume ratios, are more sensitive to neuroectodermal differentiation cues than MOs from higher cell numbers. However, MOs on DIV 14 displayed quite diverse morphological patterns based on their starting cell numbers (Figure 1(b)). MOs generated from low-cell numbers (5 × 10^2^, 1 × 10^3^, and 3 × 10^3^) showed uniform and consistent morphology containing developing neural rosettes (Figure 1(b)). In contrast, MOs formed from high-cell numbers (5 × 10^3^, 7 × 10^3^, and 1 × 10^4^) displayed relatively poor quality and uncontrolled outgrowth formation (Figure 1(b)). The expression levels of apoptosis markers increased in a starting cell number-dependent manner, lending support to our finding (Figure S1). Both forebrain (FOXG1 and LHX2) and hindbrain (HA1 and HB4) markers were suppressed in all conditions (Figure 1(d)). In general, midbrain markers (TH and LMX1B) were activated specifically in MOs generated from all numbers of starting cells (Figure 1(d)). Similarly, midbrain-specific dopaminergic (mDA) neurons expressing tyrosine hydroxylase (TH) were observed in MOs from all conditions (Figure S1). However, the expression levels of TH were relatively lower in MOs generated from high-cell numbers compared with MOs from low-cell numbers (Figure 1(d)).

To determine whether our high-throughput platform without the use of a typical orbital shaker is sufficient for producing MOs containing mature mDA neurons, we induced the maturation of MOs. Similar to MOs on DIV 14, MOs on DIV 21 that had formed from a low number of starting cells (5 × 10^2^ and 1 × 10^3^) showed a uniform morphology (Figure 1(b)). However, MOs that had formed from a high number of starting cells (3 × 10^3^, 5 × 10^3^, 7 × 10^3^, and 1 × 10^4^) were of relatively poor quality, resulting in severe outgrowths in more than 60% of MOs (Figure 1(b)) consistent with the increased expression levels of apoptotic markers and increased number of caspase 3+ cells in MOs obtained from a high number of starting cells (Figure S2). Although the size of MOs obtained from a low number of cells was significantly smaller on DIV 7, MOs from DIV 14 to 21 showed similar size regardless of the starting cell number (Figure 1(c)), indicating that the number of starting cells does not affect the growth of the resultant MOs after the maturation step. Indeed, MOs from all conditions exhibited a similar gene expression profile in which midbrain-specific markers were specifically activated at both the RNA and protein levels (Figures 1(e) and 1(f) and Figure S2), however, forebrain and hindbrain markers were not expressed in all group of MO (Figure S2). Although the numbers of TH + mDA neurons are similar in MOs from all conditions on DIV 21 (Figures 1(e) and 1(f)), we observed dramatic differences in MOs on DIV 30 (Figures 1(g) and 1(h)). We found a highly enriched and homogeneous distribution of mDA neurons in MOs derived from 5 × 10^2^ cells but a low number of mDA neurons in MOs from other conditions. This data indicate that 5 × 10^2^ cells is an optimal number of cells to start with for producing homogeneous MOs using a high-throughput platform (Figure 1(i) and Video Clip 1). For the rest of the analysis we focused on MOs generated from a starting population of 5 × 10^2^ cells and we hereafter refer to MOs from 5 × 10^2^ cells as micro MOs (*µ*MOs). Taken together, our data indicate that the generation of MOs from a low number of starting cells 5 × 10^2^ would be beneficial for both the robust specification into the midbrain and prevention of undesired differentiation into other brain regions, efficiently inhibiting the formation of both outgrowths and apoptosis level in our experimental setting using a high-throughput platform.

### 2.2. High-Speed Generation of Functionally Mature µMos

**Figure 2:**
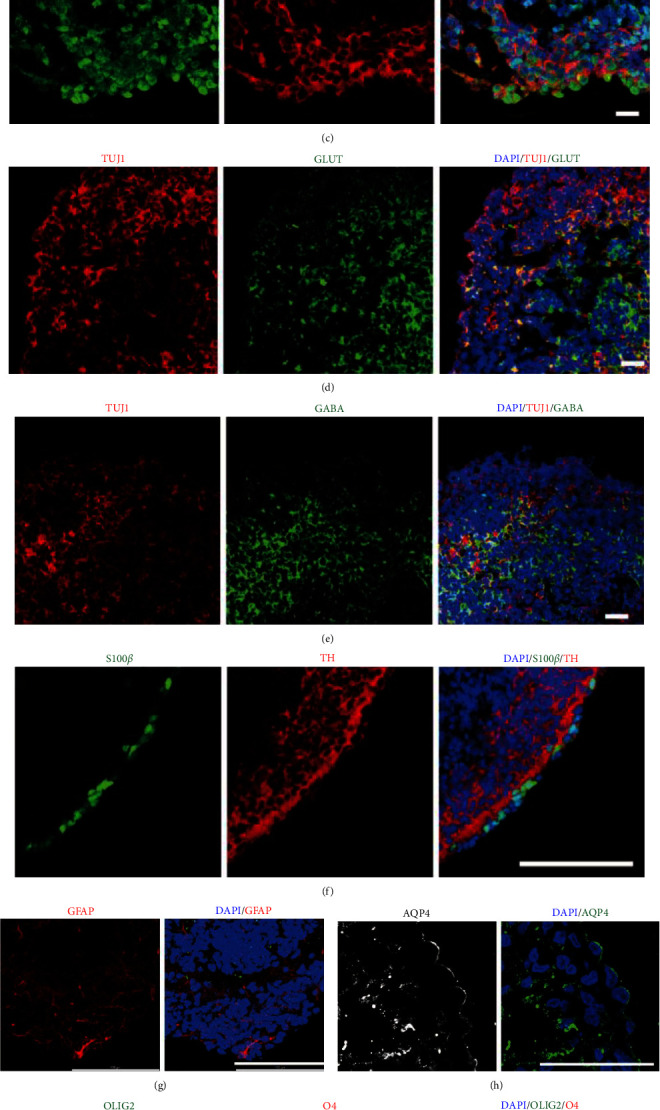
*µ*MOs show similar cell type composition to *in vivo* midbrain. (a–c) Confocal images of *µ*MOs. Scale bar, 20 *μ*m. (d and e) Confocal images of *µ*MOs showing glutamatergic neurons and GABAergic. Scale bar, 20 *μ*m. (f–h) Confocal images of *µ*MOs showing astrocyte marker S100*β* (f), GFAP (g), and AQP4 (h). Scale bar, 100 *μ*m. (i) Confocal images showing the expression pattern of oligodendrocyte markers in *µ*MOs. Scale bar, 20 *μ*m. (j) Confocal images of *µ*MOs showing mature mDA neurons. Scale bar, 20 *μ*m.

*µ*MOs homogeneously contain both early stage progenitors expressing LRTM1, SOX2 and FOXA2 (Figure 2(a)–2(c)) and mature mDA neurons expressing TH (Figure 2(c)). In contrast, *µ*MOs did not express the markers for cerebral cortex FOXG1 and TBR1 (Figure S2). Similar to typical MOs in our previous study [23], *µ*MOs also exhibited cell-type diversity. *µ*MOs contain mDA neurons, neuronal subtypes such as glutamatergic and GABAergic neurons (Figures 2(d) and 2(e)), and glial cells such as s100*β*/GFAP/AQP4+ astrocytes and OLIG2/O4+ oligodendrocytes (Figure 2(f)–2(i)). *µ*MOs were also found to contain mature mDA neurons expressing GIRK2 (Figure 2(j)). The presence of cellular diversity in *µ*MOs was also confirmed by RNA sequencing (RNAseq) analysis using *µ*MOs on DIV 30 of differentiation. *µ*MOs exhibited the elevated expression levels of multiple markers including progenitor-, midbrain-, mDA neuron-, GABAergic neuron-, glutamatergic neuron-, astrocyte-, oligodendrocyte-, and microglia-markers compared to those from undifferentiated hESCs (Figures S3 and S4). Collectively, our data indicate that the high-speed and large-scale production of *µ*MOs containing mature mDA neurons as well as other midbrain-specific cellular compartments could be achieved using a high-throughput platform.

**Figure 3:**
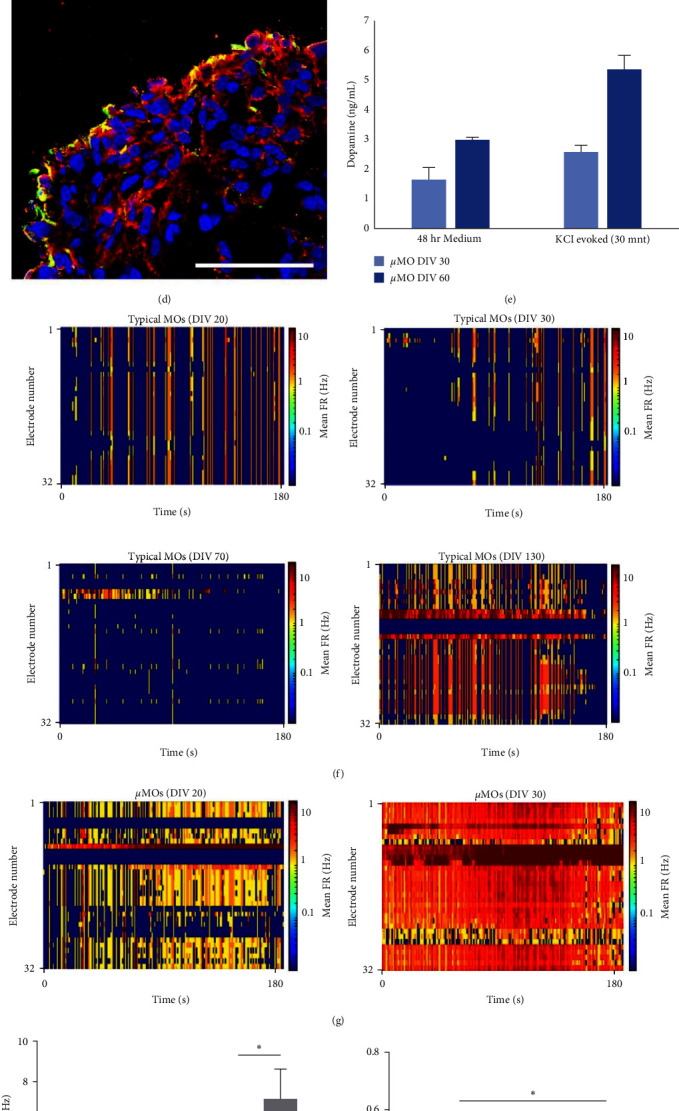
Accelerated functional maturation patterns in *µ*MOs. (a) Schematic illustration depicting the procedure for evaluating the formation of neuromelanin-like granules in *µ*MOs. (b) Accumulation of neuromelanin-like granules in *µ*MOs (DIV 30). The neuromelanin-like granules were uniformly formed in all dopamine-treated *µ*MOs (left) but not in untreated *µ*MOs (right). The formation of neuromelanin-like granules was observed on the outside of *µ*MOs. Scale bar, 100 *μ*m. (c) Fontana–Masson staining of *µ*MOs (DIV 30). Cerebral organoid was used as a negative control. Scale bar, 100 *μ*m. (d) Confocal images showing the expression pattern of dopamine in *µ*MOs (DIV 30). Scale bar, 20 *μ*m. (e) Quantification of dopamine release in *µ*MOs (DIV 30 and 60) mnt, minutes. (f, g) Color-mapped raster plots showing neural signals recorded from a 32-channel neural probe in typical MOs (f) on DIV 20, 30, 70, and 130 and *µ*MOs (g) on DIV 20 and 30. (h) Bar graph displaying mean firing rate of neural signals in typical MOs and *µ*MOs on DIV 20 and 30 (*n* = 5 independent samples for typical MOs, *n* = 4 independent samples for *µ*MOs on DIV 20, and *n* = 3 independent samples for *µ*MOs on DIV 30). Data are presented as mean ± SEM. (i) Bar graph displaying synchronization between neural signals recorded from each electrode in *µ*MOs on DIV 20 and 30 (*n* = 4 independent samples for *µ*MOs on DIV 20 and *n* = 3 independent samples for *µ*MOs on DIV 30). Data are presented as mean ± SEM. (j) Bar graph displaying the total number of connected electrodes in *µ*MOs on DIV 20 and 30 (*n* = 4 independent samples for *µ*MOs on DIV 20 and *n* = 3 independent samples for *µ*MOs on DIV 30). Data are presented as mean ± SEM.

The presence of neuromelanin-like granules accumulating in the substantia nigra pars compacta in the human midbrain is a major criterion for assessing the functional maturation level of MOs [15, 24]. Although we previously described the homogeneous distribution of neuromelanin-like granules in our MOs [23], in general, the accumulation of neuromelanin-like granules was only partially observed in a limited area in MOs even after a long-term maturation phase, i.e., 70 DIV [23] and 80 DIV [15] of differentiation. Thus, we next assessed whether *µ*MOs after only 30 DIV of differentiation could already show the accumulation of neuromelanin-like granules in the presence or absence of dopamine (Figure 3(a)). For this, we treated *µ*MOs on one half of a 96-well plate with dopamine for 10 days and omitted dopamine for the other half. To our surprise, we readily observed the accumulation of neuromelanin-like granules on the outside of *µ*MOs, indicating that mDA neurons might secrete neuromelanin-like granules (Figure 3(b)). All the dopamine-treated *µ*MOs showed the production of neuromelanin-like granules and individual *µ*MOs displayed uniform accumulation patterns in which virtually 100% of *µ*MOs successfully produced neuromelanin-like granules in a similar manner, suggesting the functional homogeneity of individual *µ*MOs (Figure 3(b)). Using Fontana–Masson staining, we also confirmed the presence of neuromelanin-like granules in *µ*MOs but not in cerebral organoids (COs) that had been matured during the same period (Figure 3(c)). Notably, *µ*MOs that were not treated with dopamine also rarely exhibited Fontana–Masson-positive granules (Figure 3(c)). As neuromelanin-like granules are rarely observed in typical MOs even after a long-term maturation step (usually 70–80 DIV after differentiation) [15, 23], we concluded that the functional maturation of *µ*MOs is much faster than that of typical MOs. Furthermore, we also confirmed the production of dopamine in *µ*MOs after only 30 days of differentiation, which is typically observed at 96–140 days of differentiation using previously described protocols [23] (Figure 3(d)), indicating that *µ*MOs could functionally mature within 30 DIV of differentiation. Similarly, dopamine release could be readily observed in *µ*MOs at DIV 30, although its level was further increased in *µ*MOs at DIV 60 (Figure 3(e)). Taken together, our data indicate that our strategy for producing *µ*MOs accelerates the functional maturation process, resulting in the high-speed production of *µ*MOs that are functionally mature and homogeneous.

To further explore the functional maturation level of *µ*MOs, we next performed electrophysical analysis. For this, we measured the electrophysiological activities of *µ*MOs (on DIV 20 and 30 of differentiation) and typical MOs (on DIV 20, 30, 70, and 130 of differentiation) in a time-course manner using a 32-channel neural probe [25]. Typical MOs from DIV 20, 30, and 70 barely displayed action potentials (Figure 3(f)), indicating that an extra long-term maturation step is essential for the functional maturation of typical MOs. In contrast, *µ*MOs from DIV 20 already exhibited higher firing rates compared with typical MOs (Figures 3(g) and 3(h)). To our surprise, *µ*MOs from DIV 30 showed a dramatically increased firing rate compared with *µ*MOs from DIV 20 (Figures 3(g) and 3(h), Figure S5). This data indicate that the number of synapses in *µ*MOs is substantially increased within 30 DIV of differentiation. Furthermore, *µ*MOs from DIV 30 also showed increased burst activity and burst duration compared with *µ*MOs from DIV 20 (Figure S5), also indicating that the neural network of *µ*MOs could be further strengthened by the increased number of synapses within 30 DIV of differentiation. By analyzing signal synchronization and neural network of *µ*MOs from DIV 20 to 30 (Figures 3(i) and 3(j)), we also found that *µ*MOs from DIV 30 show significantly strong neural network formation compared with *µ*MOs from DIV 20 (Figure S5). Collectively, *µ*MOs are functionally mature, and their functional maturation could be robustly achieved within only 30 DIV of differentiation.

### 2.3. Large-Scale Production of Uniform µMOs with Minimal Variability

**Figure 4:**
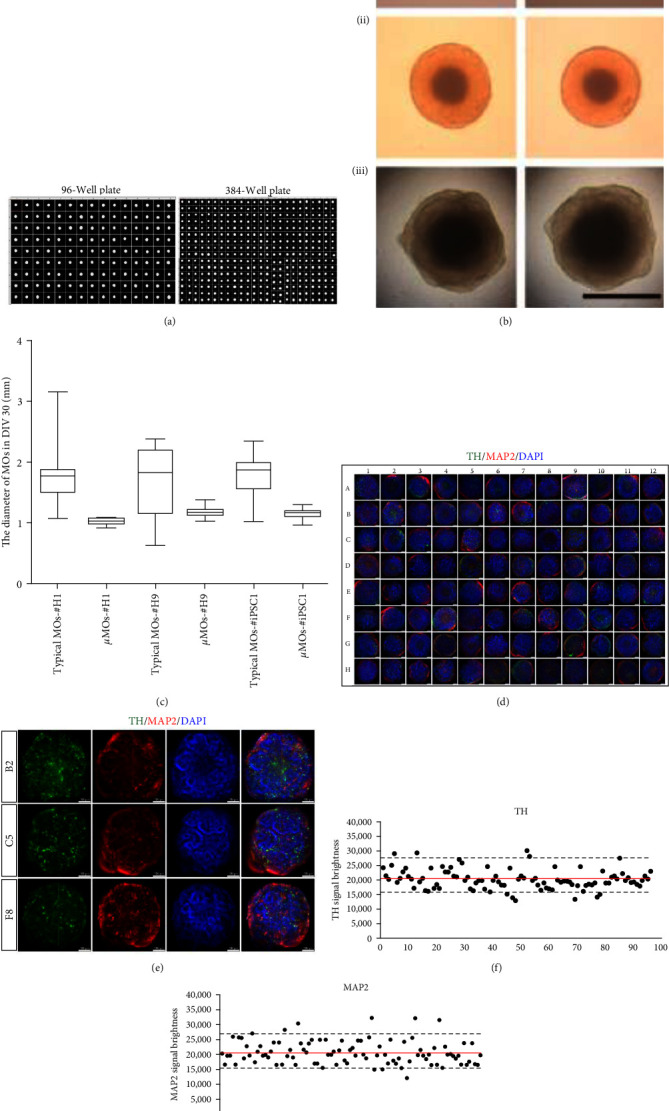
Minimal variability in *µ*MOs within and across batches and obtained from different cell lines. (a) Images of individual *µ*MOs produced in 96- (left) and 384-well plates (right) using GFP-labelled hESCs. (b) Representative morphological images of typical MOs (i) and *µ*MOs derived from 96- (ii) to 384-well (iii) plates. Scale bar, 1 mm. (c) Average diameter of typical MOs and *µ*MOs from H1 hESCs, H9 hESCs, and hiPSCs (DIV 30), 20 typical MOs or *µ*MOs from each cell line were used for analysis. Data are presented as mean ± SD from three independent experiments. (d) Overview of high-content imaging showing the expression patterns of TH and MAP2 in 96 individual *µ*MOs from 96-well plate. Scale bar, 100 *μ*m. (e) Representative examples of high-content imaging. Scale bar, 100 *μ*m. (f and g) Brightness of TH (f) and MAP2 (g) signals in 96 individual *µ*MOs. The continuous line represents the mean value of 96 individual *µ*MOs from a 96-well plate and the dotted lines show the range of SD.

Variability in organoids within and across batches and obtained from different cell lines is a major roadblock for translating brain organoid technology into the clinic [16]. As the instability of brain organoids has the potential to impede the application of brain organoids for *in vitro* disease modeling and library-scale drug screening, a standardized and reproducible approach is required for producing uniform brain organoids of consistent quality on an industrial scale [16, 17]. In the current study, we described the high-speed and large-scale production of functionally mature *µ*MOs containing mature mDA neurons using a high-throughput platform. We then sought to address the potential variability among individual *µ*MOs from distinct cell lines and batches. Specifically, we explored the consistency of individual *µ*MOs produced in a 96-well plate. We first produced *µ*MOs from H9 hESCs labeled with GFP and then assessed their morphological consistency by measuring the shape and size of individual *µ*MOs using a high-content imaging system (Figure 4(a)). We observed a uniform morphology in virtually 100% of 96 well- and 384 well-derived individual *µ*MOs (Figure 4(a)). Moreover, *µ*MOs exhibited a relatively uniform size, ranging from 0.9 to 1.3 mm (Figure 4(c)). In contrast, typical MOs showed relatively inconsistent quality, with heterogeneous morphology and size (Figures 4(b) and 4(c), Figure S6). To measure the qualitative consistency of *µ*MOs at the molecular level, we first isolated mRNA from 96 individual *µ*MOs from a 96-well plate and compared their gene expression pattern. We used typical MOs of three different classes of quality (high, medium, and low) based on their morphological differences as controls (Figure S6). As expected, typical MOs of different qualities displayed distinct transcriptional levels of two representative midbrain markers, TH and ASCL1 (Figure S6). To our surprise, the vast majority of *µ*MOs exhibited higher expression levels of TH and ASCL1 compared with typical MOs with high quality (92.7% for TH and 94.7% for ASCL1) (Figure S6).

We next performed high-content imaging of 96 individual *µ*MOs from a 96-well plate to address the homogeneous expression of TH and MAP2 at the protein level (Figure 4(d)). Using whole-mount imaging of the 96 *µ*MOs in which the abundant neurites expressing both TH and MAP2 are clearly visible (Figures 4(d) and 4(e)), we compared the mean brightness of TH and MAP2 signals from each *µ*MO (Figures 4(f) and 4(g)). When we did this, we achieved consistent signals of TH and MAP2 in individual *µ*MOs, with 87% and 81% of *µ*MOs exhibiting a similar range of TH and MAP2 brightness, respectively, or even higher than average signals (Figures 4(f) and 4(g)). Taken together, our data indicate that there is minimal inter-organoid variability with our *µ*MO strategy.

Next, we sought to address the cell line variability in the production of *µ*MOs. To this end, we produced both *µ*MOs and typical MOs from another hESCs (H9) and two lines of our homemade hiPSCs (lines 1 and 2). Compared with typical MOs, *µ*MOs generated from different cell lines showed consistent morphology and size (Figure S6). Moreover, *µ*MOs generated from different cell lines also exhibited higher expression levels of midbrain markers (Figure S6). We also assessed the batch-to-batch variability in *µ*MO generation. For this, we produced *µ*MOs from three independent experiments (H1 hESCs) and compared their morphology and size. *µ*MOs from all three independent batches exhibited uniform morphology and size on DIV 1, 7, 14, 21, and 30 (Figure S6). Furthermore, our RNAseq analysis using *µ*MOs from three independent batches display nearly indistinguishable gene expression profiles of multiple markers examined (Figures S3 and S4). Collectively, our data indicate that our novel strategy might serve as a universal platform for producing highly uniform MOs with minimal variability among organoids within and across batches and obtained from different cell lines.

### 2.4. Highly Consistent µMOs Facilitate µMO-Based In Vitro Toxicity Assay

Finally, to assess whether our *µ*MOs could serve as a drug-screening platform, we evaluated the potential of *µ*MOs as an *in vitro* high-throughput toxicity assay platform. To this end, we treated *µ*MOs with 1-methyl-4-phenyl-1,2,3,6-tetrahydropyridine (MPTP), a well-known neurotoxin and determined the its effect on *µ*MO morphology, size, and gene expression profile. We treated DIV 30 *µ*MOs for 10 days with different concentrations of MPTP (150, 300, and 500 *µ*M). The MPTP-treated *µ*MOs exhibited clear size reduction in a dose-dependent manner (Figure S7), which could be explained by the increased expression levels of apoptosis-related markers upon MPTP treatment (Figure S7). In contrast, the expression levels of midbrain markers TH and ASCL1 were suppressed upon MPTP treatment, also in a dose-dependent manner (Figure S7). MPTP-mediated cell death could also be confirmed in a dose-dependent manner by the gradually increased number of TUNEL + cells (Figure S7).

**Figure 5:**
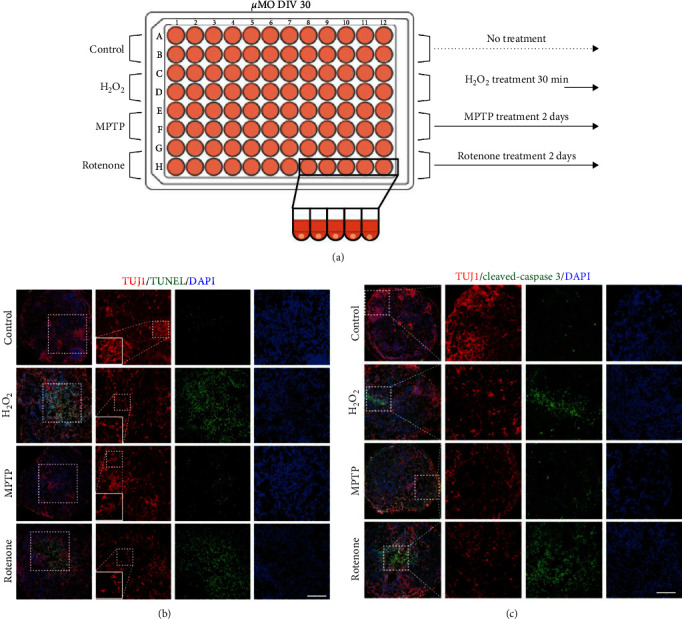
*In vitro* high-throughput toxicity assay using *µ*MOs. (a) Schematic illustration depicting the *in vitro* toxicity assay using *µ*MOs. (b and c) Confocal images showing the presence of TUNEL and cleaved-caspase 3 signals in *µ*MOs after treatment with H_2_O_2_, MPTP, and rotenone. Scale bar, 100 *μ*m.

In order to explore neurotoxin-mediated toxicity in detail, we next treated rotenone (100 *µ*M) and MPTP (150 *µ*M) on DIV30 *µ*MOs for 2 days and determined their toxicity by assessing cleaved-caspase 3 and TUNEL-positive cells (Figure 5(a)). As a positive control, we treated H_2_O_2_ on *µ*MOs for 30 min (Figure 5(a)). In contrast to nontreated *µ*MOs, both H_2_O_2_ and rotenone treated *µ*MOs exhibited massive cell death, as evidenced by the increased numbers of both cleaved-caspase 3 and TUNEL-positive cells (Figures 5(b) and 5(c)). As expected, MPTP-treated *µ*MOs exhibited the relatively mild toxicity compared with both H_2_O_2_ and rotenone treated *µ*MOs (Figures 5(b) and 5(c)) because MPTP exerts its role as a dopaminergic neurotoxin via astrocytes which exist relatively rarely in *µ*MOs. Notably, the increase in apoptosis also destroys the morphology of the nerve, resulting in the fragmentation of the nerve structure (Figure 5(b)).

To use our *µ*MOs for *in vitro* high-throughput drug screening, including toxicity screening, individual *µ*MOs should display similar responses to drug treatment. Thus, we next investigated the response of individual *µ*MOs to MPTP treatment. For this, we treated *µ*MOs with MPTP (500 *µ*M) and assessed the drug response of randomly selected *µ*MOs (10 *µ*MOs) by counting the number of TUNEL + cells. In contrast to control *µ*MOs, MPTP-treated *µ*MOs exhibited significant increases in the number of TUNEL + cells (Figure S7). Notably, the number of TUNEL + cells in randomly selected individual *µ*MOs was in a similar range (Figure S7) (10.9% TUNEL + cells in MPTP-treated *µ*MOs), indicating that individual *µ*MOs exhibit a highly consistent response against MPTP. Taken together, our data indicate that *µ*MOs exhibit a highly consistent and dose-dependent response to neurotoxin in terms of morphology, size, gene expression pattern, and cell death, rendering *µ*MOs highly suitable for *in vitro* high-throughput toxicity screening.

## 3. Discussion

Brain organoid technology has opened up a new avenue for more precise and reliable *in vitro* disease modeling and drug screening for various neuronal diseases by closely recapitulating *in vivo* microenvironments than 2D differentiation protocols that produce mostly a singular differentiated cell type without forming the *in vivo*-like tissue architecture composed of multiple cellular components [9, 26]. However, roadblocks remain and impede the translation of brain organoid technology into the clinic or industry. Recently, we and others have successfully described advanced brain organoid technologies for overcoming some of the technical roadblocks associated with brain organoids such as: (1) low-efficiency and reproducibility [16]; (2) biased cellular composition [14, 15]; (3) necrotic dead core [20, 21]; and (4) heterogeneous structure including undesired differentiation into other tissues [18, 19] or other brain regions [27]. However, fundamental issues persist and need to be overcome before initiating industrial translation of brain organoid technology such as: (1) a lengthy protocol for producing brain organoids resulting in extremely high-production costs; (2) variability in individual organoids both within and across batches and from distinct starting cell sources, and (3) the lack of a standardized large-scale production platform.

In the current study, we describe a scalable and high-speed method for robustly generating highly uniform MOs. Using our novel strategy, we were able to generate *µ*MOs with uniform and consistent morphology, size (Figure 4(a)–4(c)), and gene expression profile (Figure 4(d)–4(g)). Furthermore, our *µ*MOs also exhibited dramatically reduced formation of outgrowths (Figure 1(b)), which is a common technical issue in brain organoid production [20, 21]. Notably, the frequently observed variability among individual MOs, distinct starting cell lines, and different batches were efficiently minimized using our *µ*MO generation method (Figure 4, Figure S6). More importantly, we could generate functionally mature *µ*MOs within only 30 DIVs of differentiation, as evidenced by the formation of neuromelanin-like granules and mature electrophysiological activity (Figure 3). Taken together, our data indicate that the high-speed and large-scale production of MOs with dramatically accelerated functional maturation could be achieved using a high-throughput platform.

A recent study described the production of uniform MOs by employing an automated workflow system, producing automated MOs (AMOs) that are highly uniform in terms of morphology and gene expression pattern [28]. However, some challenging issues remained with this previous technology, such as: (1) the requirement of a costly robotic system; (2) the absence of oligodendrocytes and the presence of an extremely rare population of GABAergic and glutamatergic neurons, and (3) the requirement for an additional predifferentiation step to produce neural precursor cells [30]. In contrast, *µ*MOs could be robustly and reproducibly generated under a typical lab setting without the need for a costly automation system. Furthermore, using our novel and robust method, we could reduce the price required for producing MOs by up to 90% compared with previously described protocols [14, 15] including ours [23]. Our *µ*MOs also displayed an *in vivo*-like cell type composition, with the abundant presence of distinct subtype neurons, such as GABAergic and glutamatergic neurons, and glial cells, such as astrocytes and oligodendrocytes. Moreover, our strategy does not require any predifferentiation step of hPSCs.

In the previous study [30], the production of uniform AMOs could be achieved using predifferentiated small molecule neural precursor cells (smNPCs) but not using undifferentiated hPSCs. Indeed, although smNPC-derived AMOs were highly homogeneous, AMOs from hPSCs were relatively heterogeneous with significant variability both within and among batches [30]. As hPSCs are a major cell source routinely used for *in vitro* disease modeling and drug screening, a user-friendly method is needed that does not necessitate a costly automation system as well as an additional predifferentiation step. In the line with this issue, *µ*MO technology could be a universal and user-friendly strategy for producing highly uniform and functionally mature MOs with minimal variability among individual MOs both within and across batches and obtained from distinct starting cell sources.

We performed *in vitro* high-throughput toxicity screening using *µ*MOs to validate the compatibility of *µ*MOs in high-throughput screening. We subjected *µ*MOs to treatment with MPTP, a nigral toxin and then assessed the morphological change of *µ*MOs as a primary readout and their transcriptional status (apoptosis and midbrain markers) as a secondary readout. Following treatment of *µ*MOs with MPTP, we detected a clear dose-dependent size reduction and transcriptional alteration, indicating the potential of using *µ*MO technology to achieve rapid and reliable toxicity screening. Moreover, individual *µ*MOs exhibited a similar response to MPTP treatment, suggesting that they have high-throughput screening compatibility for evaluating the *in vitro* toxicity of drugs. As functional astrocytes produce MPP+, a metabolized toxic form of MPTP, the MPTP-mediated cell death observed in *µ*MOs indicates that our *µ*MOs with functional astrocytes are highly compatible for neurotoxin-based *in vitro* disease modeling of Parkinson's disease. Collectively, our novel approach for the high-speed and large-scale production of *µ*MOs might represent an advanced platform for high-throughput *in vitro* disease modeling and drug screening for Parkinson's and other neuronal diseases.

## 4. Materials and Methods

### 4.1. Cell Culture

Human pluripotent stem cells (hPSCs), human embryonic stem cells (hESCs), and human induced pluripotent stem cells (hiPSCs)—were maintained in TeSRTM-E8TM medium (STEMCELL Technologies). For passaging hPSCs, ReLeSRTM (STEMCELL Technologies) was used according to the manufacturer's instructions.

### 4.2. Generation of Micro Midbrain Organoids

To generate micro midbrain organoids (*µ*MOs), as in our previous study [23]. In brief, hPSCs were dissociated into single cells using TrypLETM (Gibco), and different numbers of single cell-dissociated hPSCs, ranging from 1 × 10^2^ to 1 × 10^4^ cells (1 × 10^2^, 2.5 × 10^2^, 5 × 10^2^, 1 × 10^3^, 3 × 10^3^, 5 × 10^3^, 7 × 10^3^, and 1 × 10^4^) were plated on either ultralow-attachment *U*-bottom 96-well plates (Corning) or 384-well plates (Corning) for the generation of EBs using embryoid body forming medium (EBM). The EBM medium consisted of DMEM/F12 (Corning) supplemented with 20% KSR (Gibco), 1% penicillin/streptomycin (PS) (Gibco), GlutaMAXTM (Gibco), 1% NEAA (Gibco), 55 *μ*M *ß*-mercaptoethanol (Gibco), 1 *μ*g/ml of heparin (Sigma), 3% fetal bovine serum (FBS) (Seradigm), 4 ng/ml of bFGF (Peprotech), and 100 *μ*M Y-27632 (Calbiochem). At 24 hr after plating, the medium was changed to brain organoid generation medium (BGM) containing a 1 : 1 mix of DMEM/F12 (Corning) and Neurobasal Medium (Gibco) supplemented with 100x N2 supplement (Gibco), 50x B27 without vitamin A (Gibco), 1% penicillin/streptomycin (PS) (Gibco), 1% GlutaMAXTM (Gibco), 1% NEAA (Gibco), 55 *μ*M *ß*-mercaptoethanol (Gibco), and 1 *μ*g/ml of heparin (Sigma). BGM was replaced every other day for up to 9 DIVs. For early neuroectodermal commitment and mesencephalon specification (from DIVs 1 to 3), 2 *μ*M dorsomorphin (Sigma), 2 *μ*M A83-01 (Peprotech), 1 *μ*M IWP2 (Biogems), and 3 *μ*M CHIR99021 were used, as in our previous study [23]. The specification into the mesencephalic floor plate (from DIVs 4 to 7) was induced by treating EBs with 100 ng/ml of FGF8 (Peprotech) and 2 *μ*M SAG (Peprotech) on DIV 4. From DIV 7 onward, growth factor-reduced matrigel (Corning) was added directly into BGM, supplemented with 100 ng/ml of FGF8, 2 *μ*M SAG, 200 ng/ml of laminin (BD science), and 2.5 *μ*g/ml of insulin (Thermo), containing *µ*MOs at a final concentration of 1%. For the maturation of *µ*MOs, brain organoid maturation medium (BMM) was used. BMM consisted of 1 : 1 mix of DMEM/F12 (Corning) and Neurobasal Medium (Gibco) supplemented with 100x N2 supplement (Gibco), 50x B27 (Gibco), 1% penicillin/streptomycin (PS) (Gibco), 1% GlutaMAXTM (Gibco), 1% NEAA (Gibco), 55 *μ*M *ß*-mercaptoethanol (Gibco), 1 *μ*g/ml of heparin (Sigma), 10 ng/ml of BDNF (Peprotech), 10 ng/ml of GDNF (Peprotech), 200 *μ*M ascorbic acid (Peprotech), and 125 *μ*M cAMP (Peprotech). Growth factor-reduced matrigel (Corning) was added from DIV 7 to 30.

For generating typical MOs, our previously described protocol was used [23]. In brief, 1 × 10^4^ single cell-dissociated hPSCs were plated per well in ultralow-attachment *U*-bottom 96-well plates using EBM for generating EBs. At 24 hr after plating, the medium was changed to BGM. The conditions for inducing neuroectodermal commitment, mesencephalon specification, and mesencephalic floor plate specification during the procedure for generating *µ*MOs were used for producing typical MOs. Typical MOs were embedded into growth factor-reduced matrigel (Corning), and matrigel-embedded typical MOs were transferred onto 6-cm petri dishes containing BGM supplemented with 100 ng/ml of FGF8, 2 *μ*M SAG, 200 ng/ml of laminin, and 2.5 *μ*g/ml of insulin for facilitating basal-apical lamination. From this maturation step, MOs were cultured using BMM on an orbital shaker (Stuart). BMM was replaced every other DIV. A detailed description of stem cell culture, gene expression analysis, cryosection, immunohistochemistry, Fontana–Masson staining, electrophysiology, whole mount staining and high-content imaging, i*n vitro* high-throughput screening of drug toxicity, TUNEL assay, morphological image analysis, is provided in supplemental experimental procedures.

### 4.3. Generation of Cerebral Organoids

To generate COs, 1 × 10^4^ single cell-dissociated hESCs were plated on each well of ultralow-attachment *U*-bottom 96-well plates (Corning). At 24 hr after plating, BGM supplemented with 2 *μ*M dorsomorphin and 2 *μ*M A83-01 were added to EBs. The medium was replaced every other day. After 7 days of differentiation, COs were embedded into growth factor-reduced matrigel (Corning) droplets. Matrigel-embedded COs were transferred to 6-cm petri dishes containing BGM supplemented with 200 ng/ml laminin and 2.5 *μ*g/ml insulin for inducing basal-apical lamination. After 2 days, COs were transferred into ultralow-attachment 6-well plates (Corning) containing BMM and cultured on an orbital shaker (Stuart). The culture medium was replaced every other day.

### 4.4. Generation of Hindbrain Organoids

To generate hindbrain organoids (HOs), 1 × 10^4^ single cell-dissociated hESCs were plated on each well of ultralow-attachment *U*-bottom 96-well plates (Corning). At 24 hr after plating, BGM supplemented with 2 *μ*M dorsomorphin, 2 *μ*M A83-01, and 4.25 *μ*M CHIR99021was added to EBs. The medium was replaced every other day. After 7 days of differentiation, HOs were embedded into growth factor-reduced matrigel (Corning) droplets. Matrigel-embedded HOs were transferred to 6-cm petri dishes. After 2 days, HOs were transferred into ultralow-attachment 6-well plates (Corning) containing BMM and cultured on an orbital shaker (Stuart). The culture medium was replaced every other day.

### 4.5. Gene Expression Analysis

For comparing the relative gene expression levels of markers, total RNA from organoids was isolated using the Hybrid-RTM RNA isolation kit (GeneAll). Complementary DNA (cDNA) was synthesized using the High CapaCity cDNA Reverse Transcription kit (Applied biosystems). Quantitative RT-PCR (qPCR) was carried out with the SYBR Green PCR Master Mix (Applied Biosystems) using the ABI 7500 real-time PCR system (Applied biosystems). *Δ*Ct values were calculated by subtracting the Gapdh Ct value from that of each marker gene. Relative expression levels were calculated by using 2^−*ΔΔ*Ct^ methods. Primer sequences used for qPCR are listed in Table S1.

### 4.6. Cryosection

The organoids were fixed with 4% paraformaldehyde (Sigma) for 15 min at room temperature and washed three times with PBS. The fixed organoids were transferred to 30% sucrose solution and incubated at 4°C until the organoids sank to the bottom. Equilibrated organoids were then transferred to a freezing mold containing a 1 : 1 mixture of OCT compound and 30% sucrose solution and slowly frozen on dry ice. Frozen blocks of organoids were sliced with a cryotome. The microsections of frozen blocks were attached to silane-coated glass microslides (Muto pure chemical). Sliced tissue sections were air dried.

### 4.7. Immunohistochemistry

The dried tissue sections were permeabilized and blocked with DPBS (Welgene) containing 0.03% Triton X-100 (Sigma) and 5% FBS (Seradigm) for 1 hr at room temperature. The permeabilized tissue sections were then incubated with the appropriate primary antibody for 16 hr at 4°C, and then incubated with the appropriate secondary antibody after being washed three times with DPBS (Welgene). Counterstaining was performed with TOPRO-3 (Life Technology). Primary antibodies used for immunohistochemistry are as follows: MAP2 (Sigma, 1 : 500), TH (Abcam, 1 : 500), TH (R&D system, 1 : 500), TBR1 (Abcam, 1 : 500), TUJ1 (Biolegend, 1 : 1,000), SOX2 (R&D system, 1 : 500), FOXA2 (Abcam, 1 : 500), LRTM1 (Cusabio, 1 : 500), GIRK2 (Abcam, 1 : 500), FOXG1 (Abcam, 1 : 500), GABA (Sigma, 1 : 500), GLUT (Cusabio, 1 : 500), GFAP (CiteAb, 1 : 200), S100*β* (Abcam, 1 : 200), MBP (Abcam, 1 : 200), AQP4 (Abcam, 1 : 500), O4 (R&D system, 1 : 500), OLIG2 (Merck Millipore, 1 : 500), PSD-95 (Thermo Fisher, 1 : 300), SYP (Cusabio, 1 : 500), Dopamine (ImmuSmol SAS, 1 : 500), and caspase 3 (Cell Signaling, 1 : 500).

### 4.8. Fontana–Masson Staining

The neuromelanin pigment was stained by the FontanaMasson Stain Kit (Abcam) according to the manufacturer's instructions using cryosectioned organoids.

### 4.9. Electrophysiology

To evaluate the functional maturation level of the MOs, we used a 32-channel neural probe. The width and thickness of the probe were 220 and 20 *μ*m. The microelectrodes (14 × 14 *μ*m) were located in the probe's tip, with 30 *μ*m spacing between the electrodes. The microelectrodes were coated with black platinum through electroplating to improve the recording capacity. The average impedance of black Pt-coated microelectrodes was measured 19 ± 1 k*Ω* at 1 kHz in 0.1 M PBS. To measure neural signals in the incubator, we used a miniaturized recording structure consisting of a custom microdrive to adjust the vertical position of the probe, a PDMS chamber to load the MOs, and an acrylic enclosure to prevent rapid media evaporation [29]. After loading the MO in the center of the PDMS chamber, we slowly inserted the neural probe into the organoid using the custom microdrive. After filling the chamber with fresh medium, we put the MO inserted with the neural probe into the acrylic enclosure. We measured neural signals from the MO in the incubator. Neural signals recorded from the neural probe were processed and digitized through an RHD2132 amplifier board connected to an RHD2000 evaluation system (20 KS/s per channel, 0.3-Hz high-pass filter, 6-Hz low-pass filter, and 16-bit ADC). Neural signals were recorded for at least 3 min and from at least 3 MOs per condition.

To detect neural signals from the recorded data, we used a custom Matlab code [25]. The threshold amplitude (75 *μ*V) was set at more than three times the noise amplitude (∼25 *μ*V). Also, burst signals were analyzed using an ISIN-threshold method [30] (ISI threshold: 0.1 sec; minimum number of spikes: 3). A synchronized score between electrodes was calculated using Pyspike (https://github.com/mariomulansky/PySpike). Finally, network maps between electrodes were visualized using python-louvain (https://github.com/taynaud/python-louvain/) and visualization of 3D network map (https://github.com/Hyogeun-Shin/Visualization-of-3D-network-map/tree/v1.0.0).

### 4.10. Whole Mount Staining and High-Content Imaging

For immunostaining of whole *µ*MOs without microsectioning, whole *µ*MOs were fixed with 4% paraformaldehyde (Sigma) for 15–20 min at room temperature and washed three times with PBST containing DPBS supplemented with 0.1% Triton X-100. Fixed *µ*MOs were transferred into blocking solution containing DPBS supplemented with 6% BSA (Sigma), 0.2% Triton X-100, and 0.01% sodium azide (Biosolution) and then incubated overnight on rocking platform mixer at room temperature. The samples were incubated with primary antibodies for 48 hr on a rocking platform mixer at room temperature. After washing three times with PBST, *µ*MOs were incubated with fluorescence-labeled secondary antibody and TOPRO-3 on a rocking platform mixer for 48 hr at room temperature. After rinsing three times with PBST, 96-well plates containing *µ*MOs were imaged using a THUNDER Imager Live Cell & 3D Cell Culture & 3D Assay (Leica Microsystems). Cell imaging and visualization of optical sections in 3D were carried out on a Leica DMi8 microscope using the Thunder Imaging System (Leica Microsystems). Stacks were taken at 2 *μ*m intervals in the *z*-axis. To quantify the mean brightness of TH and MAP2 signals from each *µ*MO, the images of individual *µ*MO were analyzed using with Image J software (https://imagej.nih.gov/ij/download.html).

### 4.11. In Vitro High-Throughput Screening of Drug Toxicity

For the *in vitro* toxicity assay using *µ*MOs, 96 well-derived DIV 20 *µ*MOs were treated with different concentrations of MPTP (1-methyl-4-phenyl-1,2,3,6-tetrahydropyridine) (150, 300, and 500 *µ*M). After 10 days of drug treatment, *µ*MOs were analyzed to measure changes in their morphology and size. To assess the transcriptional alterations of drug-treated *µ*MOs, randomly selected 10 *µ*MOs were used for qPCR. The drug response of individual *µ*MOs to MPTP was determined by performing TUNEL staining.

To compare neurotoxin-mediated toxicity upon treatment of different toxins, we also treated rotenone (100 *µ*M) and MPTP (150 *µ*M) on DIV 30 *µ*MOs for 2 days and determined their toxicity by assessing cleaved-caspase 3 and TUNEL-positive cells. As a positive control, we treated H_2_O_2_ on *µ*MOs for 30 min. Nontreated *µ*MOs were used as negative control.

### 4.12. TUNEL Assay

To observe apoptotic cells in *µ*MOs, the TUNEL assay was performed using the In Situ Cell Death Detection Kit fluorescein (Roche) according to the manufacturer's protocol. In briefriefly, microsectioned *µ*MOs on glass slides were prepared for the TUNEL assay. Samples were permeabilized with permeabilization solution for 2 min on ice and washed three times with PBS. They were then placed in citrate buffer, subjected to 750-W microwave irradiation for 1 min, and then translocated to cool distilled water. Samples were immersed for 30 min in tris-HCl (0.1 M and pH 7.5), containing 3% BSA and 20% normal bovine serum at room temperature. The TUNEL reaction mixture was then added to the *µ*MO section. Organoid sections were blocked and costained with antibody raised against TUJ1. Quantification of TUNEL signals was performed by counting TUNEL-positive cells out of DAPI-positive cells using Image J software (https://imagej.nih.gov/ij/download.html).

### 4.13. RNAseq Analysis

RNA-seq data paired-end files were mapped to the human genome (UCSC and hg38) build using the RNA-seq aligner STAR (v2.5.2b). After mapping, the gene expression level was calculated as Fragments Per Kilobase Million (FPKM) by Cufflinks, the transcriptome assembly and differential expression analysis tool (v2.2.1), based on the default options. To determine clustering in gene expression, statistical analyses were performed with the heatmap.2 function (gplots package) in the statistical software R (v3.3.2).

### 4.14. Morphological Image Analysis

For morphological analysis, the images of individual organoids were taken under an inverted microscope (Olympus) and the images for quantifying the diameter and area (including dead-core areas) of organoids were analyzed using Image J (https://imagej.nih.gov/ij/download.html) software.

### 4.15. Statistical Analysis

For statistical analysis, the unpaired *t*-test was used for calculating *p* values. All the values were calculated from at least triplicate experiments, and the *p* values were presented as  ^*∗*^*p* < 0.05.

## Data Availability

The data used to support the findings of this study are available from the corresponding authors upon request.

## Supplementary Materials

Supplementary Materials Figure S1: optimizing starting cell number for *µ*MO generation. Figure S2: increased cell death in *µ*MOs generated from large numbers of starting cells. Figure S3: characterization of RNAseq data. Figure S4: expression patterns of cell type-specific markers in *µ*MOs. Figure S5: electrophysiological analysis of *µ*MOs. Figure S6: *µ*MOs with minimal variability among batches and starting cell lines. Figure S7: toxicity responses in neurotoxin-treated *µ*MOs. Table S1: primers used for qPCR. Video Clip 1: homogeneous distribution of TH-positive mDA neurons in *µ*MOs.

## References

[1] Siramshetty V. B., Nickel J., Omieczynski C., Gohlke B.-O., Drwal M. N., Preissner R. (2016). WITHDRAWN—a resource for withdrawn and discontinued drugs. *Nucleic Acids Research*.

[2] Lin J. H. (1995). Species similarities and differences in pharmacokinetics. *Drug Metabolism and Disposition*.

[3] Dragunow M. (2008). The adult human brain in preclinical drug development. *Nature Reviews Drug Discovery*.

[4] Takahashi K., Yamanaka S. (2006). Induction of pluripotent stem cells from mouse embryonic and adult fibroblast cultures by defined factors. *Cell*.

[5] Lee G., Papapetrou E. P., Kim H. (2009). Modelling pathogenesis and treatment of familial dysautonomia using patient-specific iPSCs. *Nature*.

[6] Matsa E., Burridge P. W., Yu K.-H. (2016). Transcriptome profiling of patient-specific human iPSC-cardiomyocytes predicts individual drug safety and efficacy responses in vitro. *Cell Stem Cell*.

[7] Shi Y., Inoue H., Wu J. C., Yamanaka S. (2017). Induced pluripotent stem cell technology: a decade of progress. *Nature Reviews Drug Discovery*.

[8] Yin X., Mead B. E., Safaee H., Langer R., Karp J. M., Levy O. (2016). Engineering stem cell organoids. *Cell Stem Cell*.

[9] Liu C., Oikonomopoulos A., Sayed N., Wu J. C. (2018). Modeling human diseases with induced pluripotent stem cells: from 2D to 3D and beyond. *Development*.

[10] Kim J., Koo B.-K., Knoblich J. (2020). Human organoids: model systems for human biology and medicine. *Nature Reviews Molecular Cell Biology*.

[11] Zhou J., Li C., Liu X. (2020). Infection of bat and human intestinal organoids by SARS-CoV-2. *Nature Medicine*.

[12] Luo C., Lancaster M. A., Castanon R., Nery J. R., Knoblich J. A., Ecker J. R. (2016). Cerebral organoids recapitulate epigenomic signatures of the human fetal brain. *Cell Reports*.

[13] Paşca A. M., Sloan S. A., Clarke L. E. (2015). Functional cortical neurons and astrocytes from human pluripotent stem cells in 3D culture. *Nature Methods*.

[14] Qian X., Nguyen H. N., Song M. M. (2016). Brain-region-specific organoids using mini-bioreactors for modeling ZIKV exposure. *Cell*.

[15] Jo J., Xiao Y., Sun A. X. (2016). Midbrain-like organoids from human pluripotent stem cells contain functional dopaminergic and neuromelanin-producing neurons. *Cell Stem Cell*.

[16] Kelava I., Lancaster M A. (2016). Stem cell models of human brain development. *Cell Stem Cell*.

[17] Watanabe M., Buth J. E., Vishlaghi N. (2017). Self-organized cerebral organoids with human-specific features predict effective drugs to combat Zika virus infection. *Cell Reports*.

[18] Lancaster M. A., Knoblich J. A. (2014). Generation of cerebral organoids from human pluripotent stem cells. *Nature Protocols*.

[19] Lancaster M. A., Corsini N. S., Wolfinger S. (2017). Guided self-organization and cortical plate formation in human brain organoids. *Nature Biotechnology*.

[20] Berger E., Magliaro C., Paczia N. (2018). Millifluidic culture improves human midbrain organoid vitality and differentiation. *Lab on a Chip*.

[21] Nickels S. L., Modamio J., Mendes-Pinheiro B., Monzel A. S., Betsou F., Schwamborn J. C. (2020). Reproducible generation of human midbrain organoids for *in vitro* modeling of Parkinson’s disease. *Stem Cell Research*.

[22] Monzel A. S., Smits L. M., Hemmer K. (2017). Derivation of human midbrain-specific organoids from neuroepithelial stem cells. *Stem Cell Reports*.

[23] Kwak T. H., Kang J. H., Hali S. (2020). Generation of homogeneous midbrain organoids with in vivo-like cellular composition facilitates neurotoxin-based Parkinson’s disease modeling. *Stem Cells*.

[24] Smits L. M., Reinhardt L., Reinhardt P. (2019). Modeling Parkinson’s disease in midbrain-like organoids. *npj Parkinson’s Disease*.

[25] Shin H., Son Y., Chae U. (2019). Multifunctional multi-shank neural probe for investigating and modulating long-range neural circuits in vivo. *Nature Communications*.

[26] Hale L. J., Howden S. E., Phipson B. (2018). 3D organoid-derived human glomeruli for personalised podocyte disease modelling and drug screening. *Nature Communications*.

[27] Fleck J. S., He Z., Boyle M. J., Camp J. G., Treutlein B. (2020). Resolving brain organoid heterogeneity by mapping single cell genomic data to a spatial reference. *bioRxiv*.

[28] Renner H., Grabos M., Becker K. J. (2020). A fully automated high-throughput workflow for 3D-based chemical screening in human midbrain organoids. *Elife*.

[29] Shin H., Jeong S., Lee J.-H., Sun W., Choi N., Cho I.-J. (2021). 3D high-density microelectrode array with optical stimulation and drug delivery for investigating neural circuit dynamics. *Nature Communications*.

[30] Bakkum D. J., Radivojevic M., Frey U., Franke F., Hierlemann A., Takahashi H. (2014). Parameters for burst detection. *Frontiers in Computational Neuroscience*.

