# Supplementary material for: Production of Highly Uniform Midbrain Organoids from Human Pluripotent Stem Cells
Source: Stem Cells Int. 2023 Sep 29;2023:3320211. doi: 10.1155/2023/3320211 (PMC10558263; doi:10.1155/2023/3320211)
Supplement: Supplementary Materials — Figure S1: optimizing starting cell number for µMO generation. Figure S2: increased cell death in µMOs generated from large numbers of starting cells. Figure S3: characterization of RNAseq data. Figure S4: expression patterns of cell type-specific markers in µMOs. Figure S5: electrophysiological analysis of µMOs. Figure S6: µMOs with minimal variability among batches and starting cell lines. Figure S7: toxicity responses in neurotoxin-treated µMOs. Table S1: primers used for qPCR. Video Clip 1: homogeneous distribution of TH-positive mDA neurons in µMOs. [file 3320211.f1.docx]

**­Supplementary Information**

Production of highly uniform midbrain organoids from human pluripotent stem cells

Xuerui Yao, Ji Hyun Kang, Kee-Pyo Kim, Hyogeun Shin, Zhe-Long Jin , Hao Guo, Yong-Nan Xu, Ying-Hua Li, Sai Hali, Jeongwoo Kwon, Hyeonwoo La, Chanhyeok Park, Yong-June Kim, Lin Wang, Kwonho Hong, Qilong Cao, Il-Joo Cho, Nam-Hyung Kim & Dong Wook Han

**Supplementary figures**

Supplementary Fig. 1, Supplementary Fig. 2, Supplementary Fig. 3, Supplementary Fig. 4, Supplementary Fig. 5, Supplementary Fig. 6, Supplementary Fig. 7

**Supplementary table**

Supplementary Table 1

**Information of Supplementary Video**

Supplementary Video 1

**Supplementary Figures**


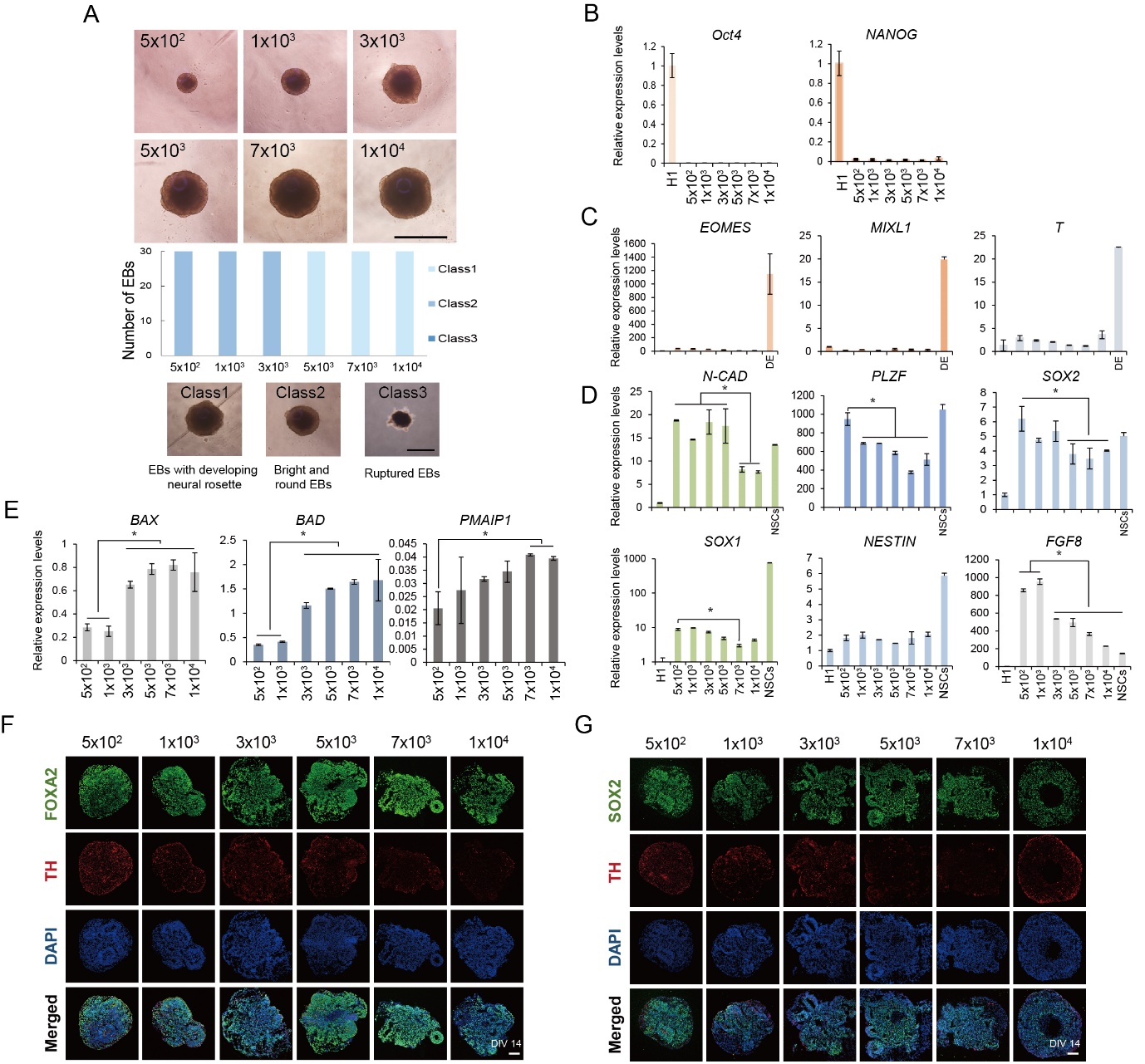


**Supplementary Fig. 1 Optimizing starting cell number for** **µMO generation.**

**(A)** The morphology and quality of MOs generated from a different number of starting cells (DIV 7). Scale bar, 1mm.

**(B**, **C**, **D)** Expression of pluripotency **(B)**, mesendoderm **(C)**, and neuroectoderm markers **(D)** was analyzed by qPCR using MOs generated from a different number of starting cells (DIV 7). Expression levels are normalized to those of undifferentiated hESCs. DE (definitive endoderm) and NSCs (neural stem cells) were used as positive controls. Data are presented as mean ± SD of triplicate values.

**(E)** Expression of apoptosis markers was analyzed by qPCR using MOs generated from a different number of starting cells (DIV 14). Data are presented as mean ± SD of triplicate values.

**(F, G)** Representative confocal images of MOs generated from a different number of starting cells (DIV 14). Scale bar, 100 μm.


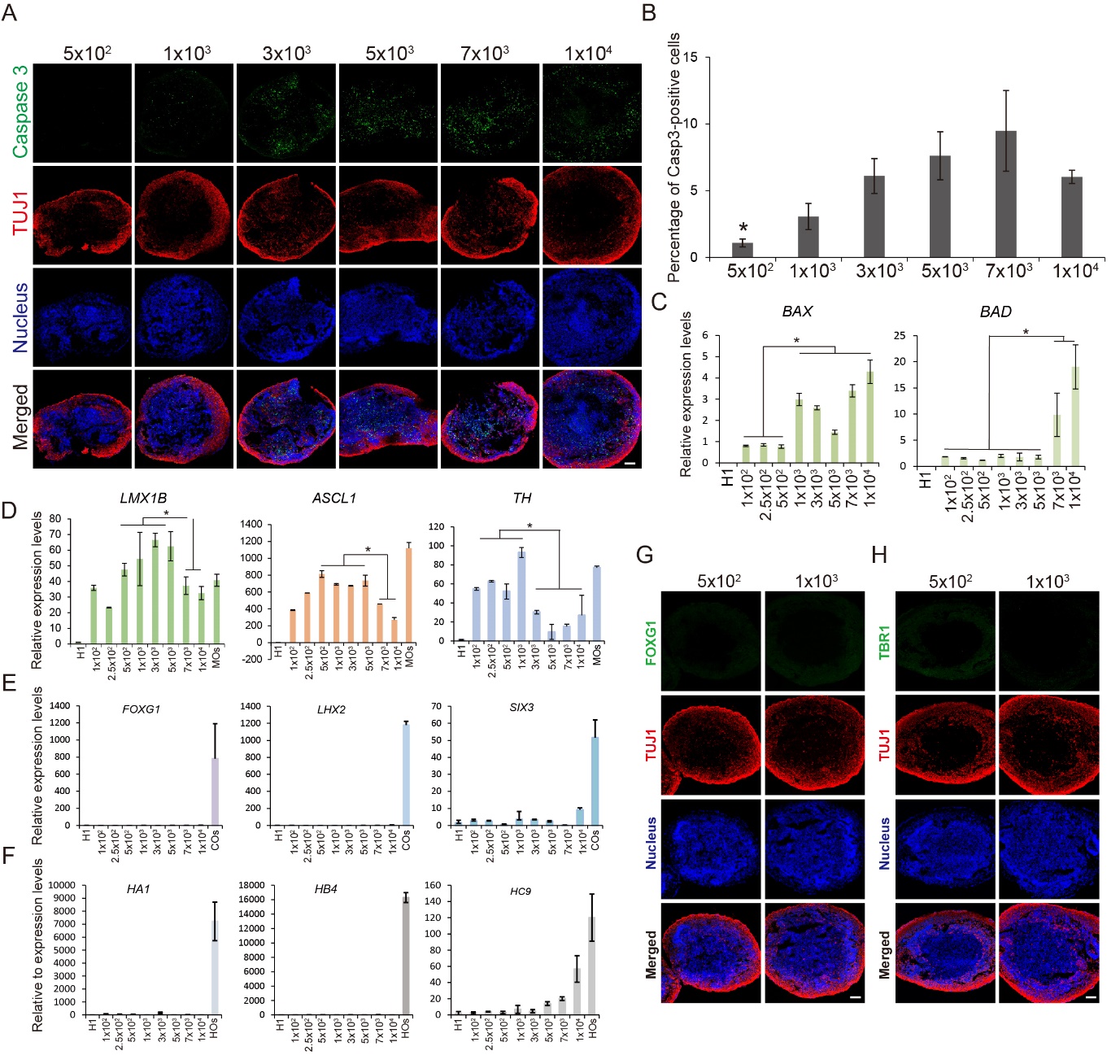


**Supplementary Fig. 2** **Increased cell death in µMOs generated from large numbers of starting cells.**

**(A)** Representative confocal images of MOs generated from a different number of starting cells expressing Caspase-3 (DIV 21). Scale bar, 100 μm.

**(B)** Percentage of Caspase-3 positive cells in µMOs generated from a different number of starting cells (DIV 21). Data are presented as mean ± SD of triplicate values.

**(C)** Expression of apoptosis markers was analyzed by qPCR using µMOs generated from a different number of starting cells (DIV 21). Expression levels are normalized to those of undifferentiated hESCs. Data are presented as mean ± SD of triplicate values.

**(D**, **E**, **F)** Expression of midbrain (**D**) forebrain **(E)** and hindbrain **(F)** markers was analyzed by qPCR using µMOs generated from a different number of starting cells (DIV 21). Expression levels are normalized to those of undifferentiated hESCs. Data are presented as mean ± SD of triplicate values.

**(G**, **H)** Confocal images showing the expression pattern of cerebral cortex markers FOXG1 **(G)** and TBR1 **(H)** in µMOs (DIV 30). Scale bar, 100 μm.

**
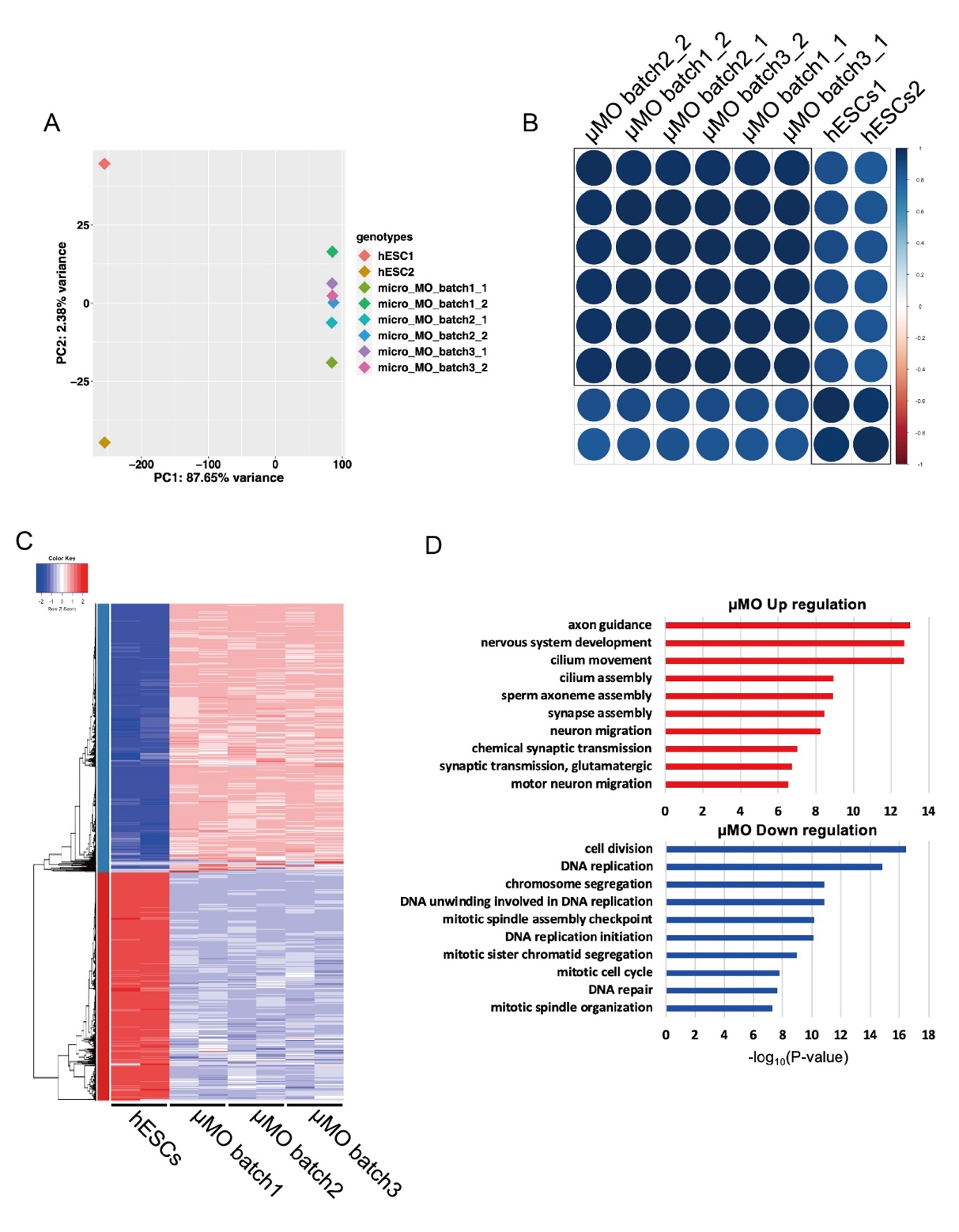
Supplementary Fig. 3 Characterization of RNAseq data.**

(**A**) PCA plot of RNA-seq data for two biological replicates.

(**B**) Correlation plot of RNA-seq data for two biological replicates.

(**C**) Heatmap illustration showing differentially expressed genes.

(**D**) Gene ontology analysis of the two groups classified in (C).


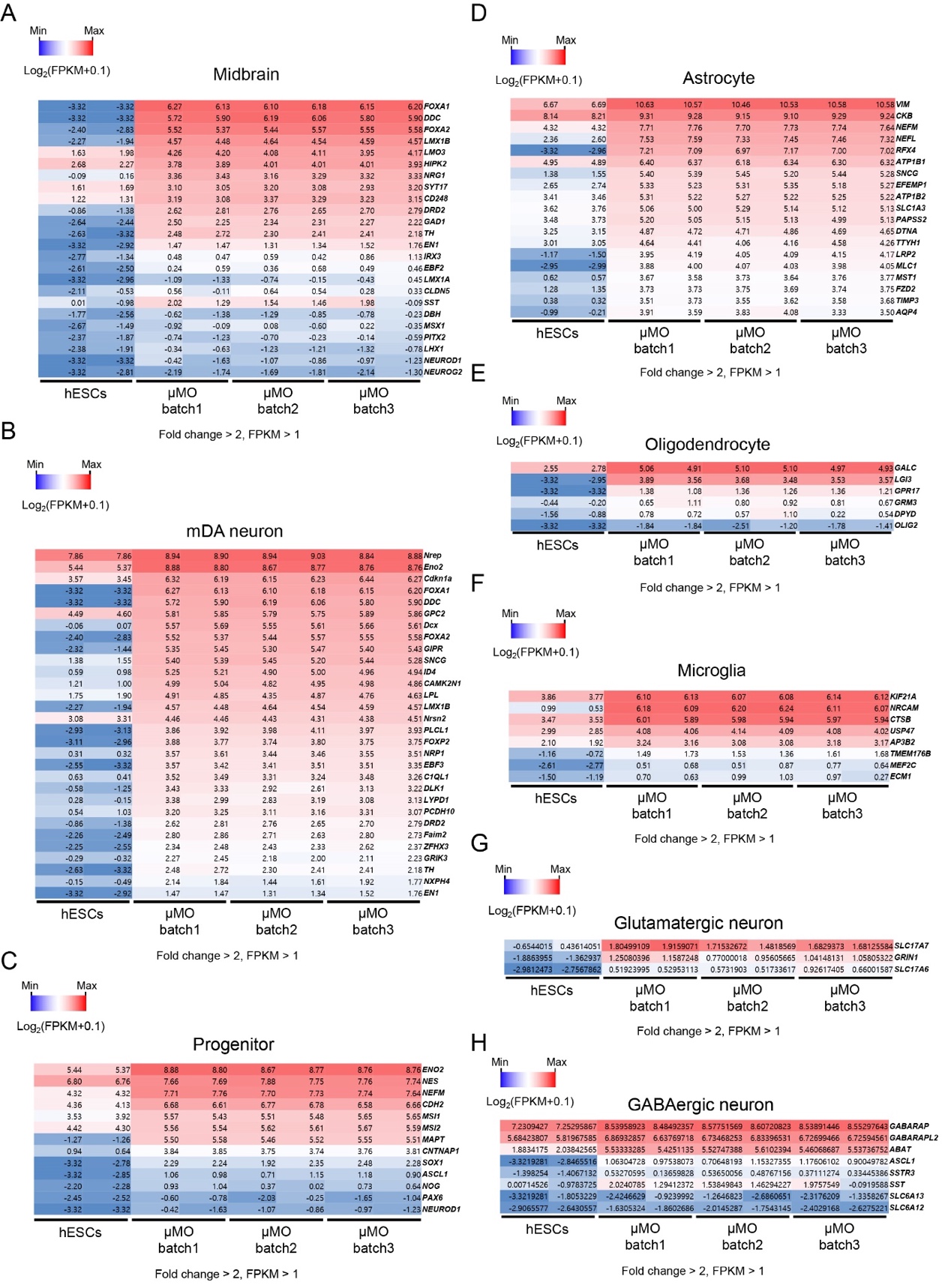


**Supplementary Fig. 4 Expression patterns of cell type-specific markers in µMOs.**

**(A**, **B**, **C**, **D**, **E**, **F**, **G**, **H)** Heatmaps representing the expression patterns of markers related to the midbrain (**A**), mDA neurons (**B**), progenitors (**C**), astrocytes (**D**), oligodendrocytes (**E**), microglia (**F**), glutamatergic neurons (**G**), and GABAergic neurons (**H**). Red and blue colors represent higher and lower expression levels, respectively.


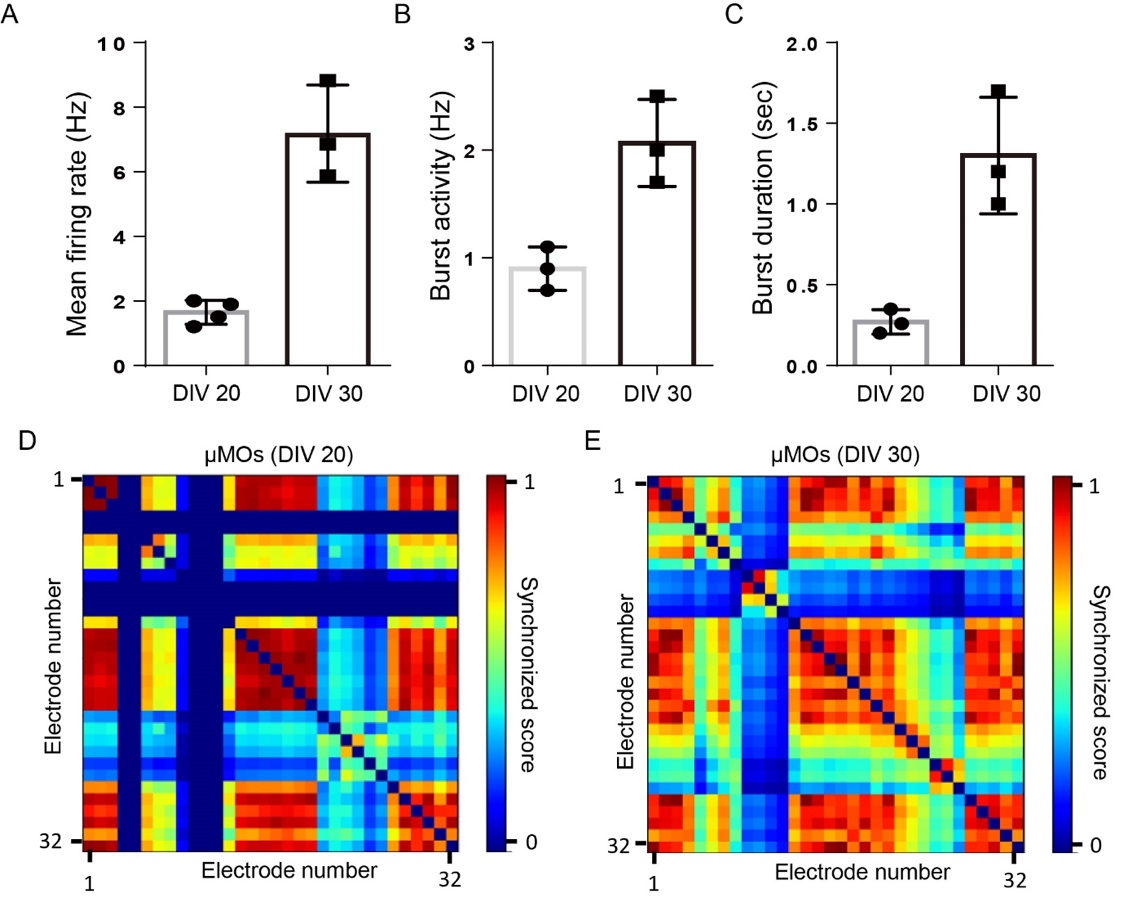


**Supplementary Fig. 5** **Electrophysiological analysis of** **µMOs.**

**(A)** Bar graph displaying mean firing rate of recorded signals in µMOs on DIV 20 and 30. (n = 4 independent samples for µMOs on DIV 20 and n = 3 independent samples for µMOs on DIV 30). Data are presented as mean ± SEM.

**(B)** Bar graph displaying burst activity of recorded signals in µMOs on DIV 20 and 30. (n = 4 independent samples for DIV 20 and n = 3 independent samples for DIV 30). Data are presented as mean ± SEM.

**(C)** Bar graph displaying burst duration of recorded signals on µMOs on DIV 20 and 30. (n = 4 independent samples for DIV 20 and n = 3 independent samples for DIV 30). Data are presented as mean ± SEM.

**(D**, **E)** Color-mapped cross-correlation matrices showing synchronized scores between electrodes based on recorded signals from µMOs on DIV 20 **(D)** and DIV 30 **(E)**.


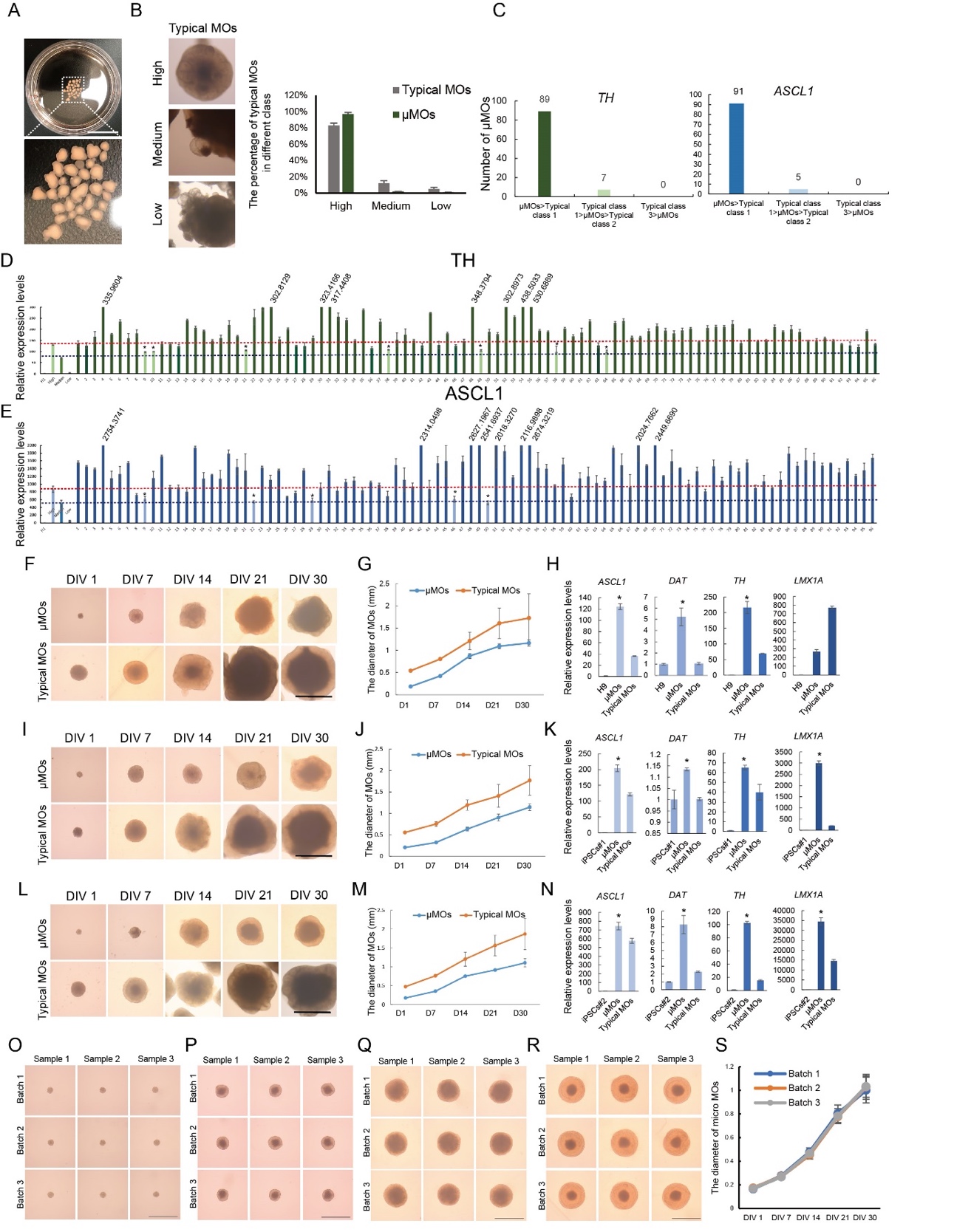


**Supplementary Fig. 6** **µMOs with minimal variability among batches and starting cell lines**

**(A)** Heterogeneous morphology and size of typical MOs (DIV 30). Scale bar, 1 cm.

**(B)** The percentage (right) of typical MOs corresponding to high-, medium-, and low-quality class based on their morphological differences (left). Data are presented as mean ± SD from three independent experiments.

**(C)** The qualitative consistency of individual µMOs was analyzed by counting the number of µMOs showing higher expression levels of *TH* and *ASCL1* compared with high-quality typical MOs. The number of µMOs showing relatively lower expression levels compared with high-quality typical MOs but higher expression levels compared with medium-quality typical MOs.

**(D, E)** Expression patterns of *TH* **(D)** and *ASCL1* **(E)** in individual µMOs from a 96-well plate (day 30) were compared with those of typical MOs of high-, medium-, and low-quality using qPCR. Expression levels are normalized to those of undifferentiated hESCs. Data are presented as mean ± SD of triplicate values.

**(F**, **I, L)** Representative morphological images of µMOs and typical MOs derived from H9 hESCs **(F)**, hiPSC line#1 **(I)**, and hiPSC line#2 **(L)** at different time points. Scale bar, 1 mm.

**(G**, **J**, **M)** The average diameter of µMOs and typical MOs derived from H9 hESCs **(G)**, hiPSC line#1 **(J)**, and hiPSC line#2 **(M)** at different time points. Data are presented as mean ± SD from three independent experiments.

**(H**, **K**, **N)** Expression levels of midbrain markers were compared between µMOs and typical MOs derived from H9 hESCs **(H)**, hiPSC line#1 **(K)**, and hiPSC line#2 **(N)** using qPCR. Expression levels are normalized to those of undifferentiated hPSCs. Data are presented as mean ± SD of triplicate values.

**(O**, **P**, **Q**, **R)** Representative morphological images of µMOs from three independent batches at different time points **(O**: DIV 1, **P**: DIV 7, **Q**: DIV 14, and **R**: DIV21**)**. Scale bar, 1 mm.

**(S)** Average diameter of µMOs from three independent batches (DIV 30). Data are presented as mean ± SD from three independent experiments (n=20 for each batch).


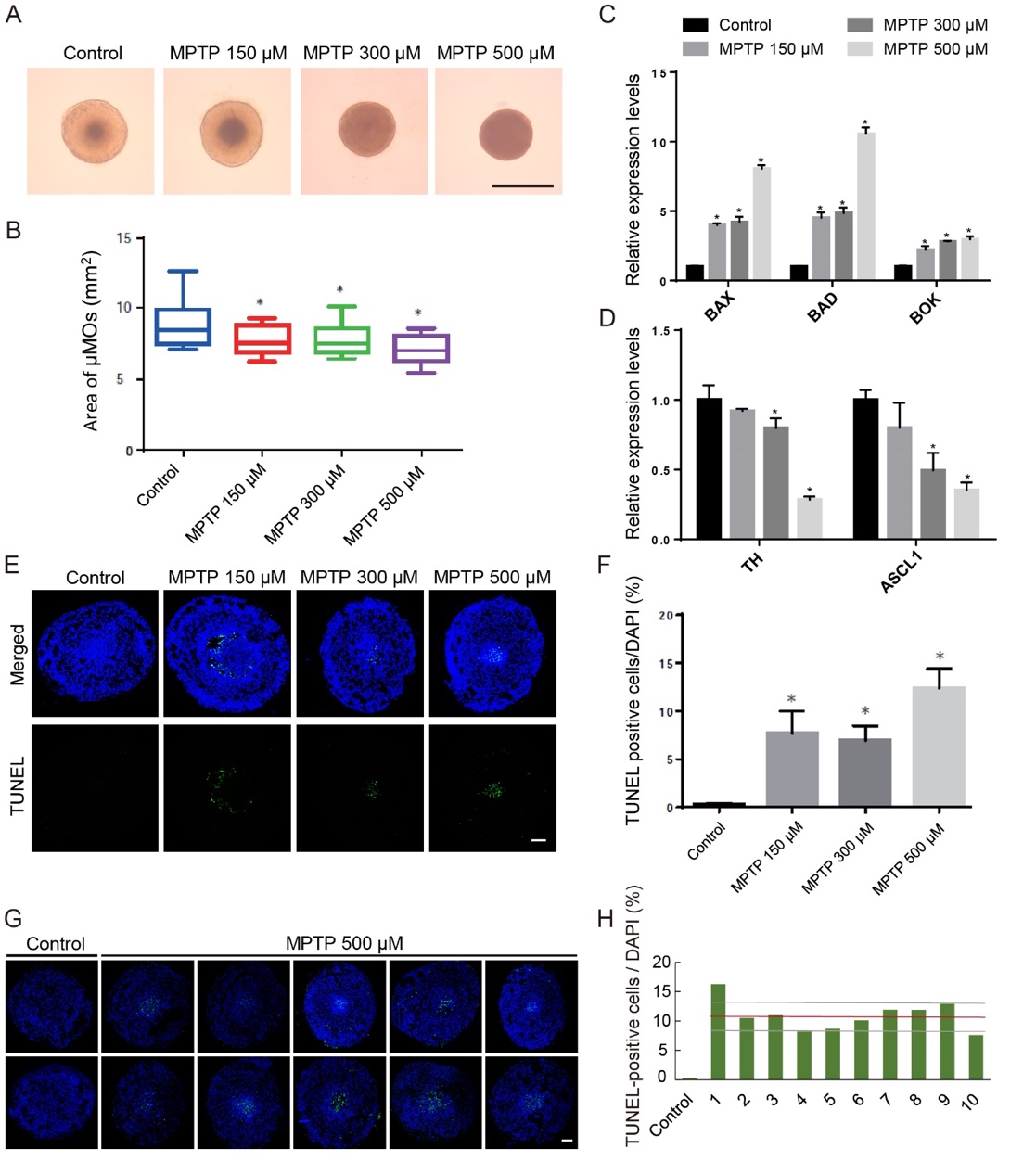


**Supplementary Fig. 7** **Toxicity responses in neurotoxin-treated µMOs.**

**(A)** The morphological changes of µMOs after treatment with different concentrations of MPTP. Scale bar, 1 mm.

**(B)** The change of µMO size after treatment with different concentrations of MPTP. Data are presented as mean ± SD from three independent experiments.

**(C, D)** Expression levels of apoptosis **(C)** and midbrain markers **(D)** in MPTP-treated µMOs were analyzed using qPCR. Expression levels are normalized to those of untreated µMOs. Data are presented as mean ± SD of triplicate values.

**(E)** Representative confocal images showing the presence of TUNEL-labelled cells in µMOs after treatment with different concentrations of MPTP**.** Scale bar, 100 μm.

**(F)** Percentage of TUNEL-positive cells in µMOs after treatment with different concentrations of MPTP. Data are presented as mean ± SD from three independent experiments.

**(G)** Confocal images showing the presence of TUNEL-labelled cells in 10 randomly selected individual µMOs after treatment with MPTP. Scale bar, 100 μm.

**(H)** Percentage of TUNEL-positive cells in 10 randomly selected individual µMOs after treatment MPTP.

**Supplementary Table**

**Supplementary Table 1. Primers used for qPCR**

| **Gene Name** | **Genebank Number** | **Primer sequence** |
| --- | --- | --- |
| *NANOG* | AB093576.1 | TGCAACCTGAAGACGTGTGA |
|  |  | CTATGAGGGATGGGAGGA |
| *OCT4* | BC117435.1 | GACAGGGGGAGGGGAGGAGCTAGG |
|  |  | CTTCCCTCCAACCAGTTGCCCCAAAC |
| *SOX2* | BC013923.2 | AGACTGCACATGAGCCAGCA |
|  |  | CGTCTCCAGCCAGCTTCAAC |
| *N-CAD* | AF006514.1 | TGATGAAGAAGGTGGAGGAGAAGA |
|  |  | ATTCGTCGGATTCCCACAGG |
| *PLZF* | NM_006006.5 | TCCCGCCCGACTGGAGGATA |
|  |  | TTCTTTCCTGGCTCCCCGCTC |
| *EOMES* | AB031038.1 | CTCAAAAGGCATGGGAGGGTA |
|  |  | CACCACCAAGTCCATCTGCAA |
| *T* | BC098425.1 | CGCCTCATAGCCTCATGGAC |
|  |  | CACTGGCTGCCACGACAAA |
| *BOK* | AF089746.1 | GCTGGCCACATCTTCTCTGC |
|  |  | TGCTGACCACACACTTGAGGAC |
| *BAX* | NM_007527 | TGCTGACGTGGACACGGACT |
|  |  | CCAGCCACCCTGGTCTTGGA |
| *BAD* | NM_007522 | TCGGAGTCGCCACAGTTCGT |
|  |  | GCGCTCTTTGGGCGAGGAAG |
| *PMAIP1* | KU178264.1 | GGGAAGAAGGCGCGCAAGAA |
|  |  | AGTTTCTGCCGGAAGTTCAGTTTGT |
| *FOXG1* | BC050072 | GCGGGCCAGACCAGTTACTT |
|  |  | CCCAGACAGTCCCGTCGTAA |
| *LHX2* | AF124735.1 | ACTTCTGTGCCTGGCAACCTG |
|  |  | TCTGTTTCCAGGCGAGATCCT |
| *SIX3* | AF049339.1 | AACCTCCAGCGACTCGGAAT |
|  |  | TTCGGTTTGTTCTGGGGATG |
| *DAT* | NM_001044.5 | CGCCACTGGCTCAAGGTGTA |
|  |  | CCGGCACGGAAAGGTGTAA |
| *TH* | NM_199292.2 | CTGAGATTCGGGCCTTCGAC |
|  |  | TGCACCTAGCCAATGGCACT |
| *LMX1B* | *AH006310.2* | GGCATCAAGATGGAGGAGCA |
|  |  | TGGTGAGGGCTTGCTGACAC |
| *LMX1A* | NM_177398.4 | AAAGCGCGATCGACACCTC |
|  |  | TCCCGGTAGAAGCAGGTGGT |
| *ASCL1* | NM_004316.4 | GGTGATCGCACAACCTGCAT |
|  |  | GTTCTGAGCGCTTCCCGTTT |
| *HOXC9* | AH010088.2 | CTCCTAGCGTCCAGGTTTCC |
|  |  | CGGGTGATATACCACGGACG |
| *HOXA1* | U10421.1 | AAATCAGGAAGCAGACCCAC |
|  |  | GTAGCCGTACTCTCCAACTTTC |
| *HOXB4* | AF307160.1 | TACCCCTGGATGCGCAAAGTTC |
|  |  | TGGTGTTGGGCAACTTGTGG |
| *MIXL1* | [NM_001282402.2](https://www.ncbi.nlm.nih.gov/nuccore/NM_001282402.2) | GGTACCCCGACATCCACTTG |
|  |  | ATACCTGGAAGAGGGGAGAAA |
| *SOX1* | [NM_005986.3](https://www.ncbi.nlm.nih.gov/entrez/viewer.fcgi?db=nucleotide&id=1653960793) | GCCGAGTGGAAGGTCATGTC |
|  |  | TTCTTGAGCAGCGTCTTGGTC |
| *NESTIN* | [NM_006617.2](https://www.ncbi.nlm.nih.gov/entrez/viewer.fcgi?db=nucleotide&id=1519243543) | AGGAAAAGACCATCTGCCCG |
|  |  | GCCTCTCAGCCAGAAACCAT |
| *FGF8* | [NM_001206389.2](https://www.ncbi.nlm.nih.gov/nuccore/NM_001206389.2) | CTTCGCAAAGCTCATCGTGG |
|  |  | CATGAAGTGGACCTCACGCT |
| *KROX20* | [NM_001136178.2](https://www.ncbi.nlm.nih.gov/entrez/viewer.fcgi?db=nucleotide&id=1890275397) | ACCGCCTCCTCCTCCTTATT |
|  |  | GGGTAGGCCAGAGAGGAAGA |
| *S100b* | [NM_006272.3](https://www.ncbi.nlm.nih.gov/entrez/viewer.fcgi?db=nucleotide&id=1519312286) | ACAATGATGGAGACGGCGAA |
|  |  | ACTCGTGGCAGGCAGTAGTA |
| *OLIG2* | [NM_005806.4](https://www.ncbi.nlm.nih.gov/entrez/viewer.fcgi?db=nucleotide&id=1519313490) | CCAGAGCCCGATGACCTTTT |
|  |  | TCCGGCTCTGTCATTTGCTT |
| *MBP* | [NM_002385.3](https://www.ncbi.nlm.nih.gov/entrez/viewer.fcgi?db=nucleotide&id=1676440489) | CCAGGATTTGGCTACGGAGG |
|  |  | TGGGTGATCCAGAGCGACTA |

**Supplementary Video**

**Supplementary Video 1. Homogeneous distribution of TH-positive mDA neurons in µMOs**

The video showing the global distribution of MAP2- and TH-positive cells in µMOs. The green and red colors mark the MAP2-positive neurons and TH-positive mDA neurons, respectively. The nucleus was counterstained with TOPRO-3 (blue color).
